# Do older vehicles affect occupants of all ages equally? Age-group differences in the association between vehicle age and fatal or suspected serious injury in U.S. police-reported passenger-vehicle crashes, 2016–2024

**DOI:** 10.1371/journal.pone.0362075

**Published:** 2026-11-12

**Authors:** Tianxing Ji, Mingxing Gao, Huajian Li, Haixiao Wang, Wei Zhou

**Affiliations:** 1 College of Energy and Transportation Engineering, Inner Mongolia Agricultural University, Hohhot, Inner Mongolia, China; 2 Research Institute of Highway, Ministry of Transport, Beijing, China; Tongji University, CHINA

## Abstract

Older vehicles are associated with worse crash injury outcomes, but whether this association varies across occupant age groups and effect scales remains unclear. We examined the association between vehicle age and fatal or suspected serious injury among occupants in U.S. police-reported passenger-vehicle crashes and compared age-group patterns on relative and absolute scales. We analyzed 2016–2024 National Highway Traffic Safety Administration Crash Report Sampling System data. Overall and age-group-specific adjusted odds ratios (aORs) were estimated using survey-weighted logistic regression; standardized predicted probabilities and probability differences were calculated under a common covariate distribution. The primary analysis included 899,273 occupants, of whom 46,367 sustained fatal or suspected serious injury. The survey-weighted outcome proportion increased from 0.72% in vehicles aged 0–4 years to 2.15% in those aged ≥20 years. The corresponding overall aOR was 2.34 (95% CI, 2.19–2.49). The full vehicle-age-category × occupant-age-group interaction (P = 0.032) and ≥20-versus-0–4-year age-group heterogeneity (P = 0.020) were statistically significant. For this comparison, aORs were 2.80 (95% CI, 2.27–3.44), 2.39 (2.23–2.57), 2.31 (2.07–2.57), and 1.99 (1.75–2.26) for occupants aged 0–17, 18–44, 45–64, and ≥65 years, respectively. After within-group continuous-age adjustment, the full interaction was not statistically significant (P = 0.083), whereas the ≥ 20-versus-0–4-year comparison retained significant age-group heterogeneity (P = 0.024). Occupants aged ≥65 years had the highest standardized predicted probability in every vehicle-age category. Standardized probability differences for vehicles aged ≥20 versus 0–4 years were 0.77, 0.97, 1.08, and 1.12 percentage points across the four age groups, with no statistically significant overall heterogeneity (P = 0.080). Increasing vehicle age was consistently associated with greater odds of fatal or suspected serious injury, while relative associations, standardized predicted probabilities, and absolute probability differences showed different age-group patterns. Considering these measures together provides a more complete characterization of age-group differences in vehicle-age associations.

## 1 Introduction

Road traffic injuries are a leading cause of preventable death and disability worldwide and remain a major public health burden [1]. In the United States, motor vehicle traffic fatalities declined in 2024 compared with 2023 but still totaled 39,254 [2,3]. Passenger-vehicle occupants accounted for more than half of these deaths [4]. Within the Safe System framework, reducing road traffic deaths and serious injuries requires coordinated attention to the road environment, road-user behavior, and vehicle performance in crash avoidance and occupant protection [5]. For occupants involved in crashes, the crash protection provided by vehicle structures and restraint systems is a critical determinant of injury outcomes [6–8]. As safety regulations and vehicle design have evolved, occupant protection has diverged across vehicle manufacturing cohorts [9]. Vehicle age is therefore a readily observed indicator of safety differences within the real-world fleet. The information it captures reflects both the safety-technology context of the period in which a vehicle was manufactured and changes in its in-use condition over prolonged use, providing an entry point for examining injury differences between newer and older vehicles.

Analyses by the National Highway Traffic Safety Administration (NHTSA) of national police-reported and fatal crashes found that older vehicles or earlier model years were associated with more severe occupant injury outcomes [10,11]. A case-control study from New Zealand and data from the U.S. Crash Injury Research and Engineering Network produced similar findings [12,13]. A 2024 study of adult occupants in New York State further found that earlier vehicle model years were associated with higher odds of serious injury or death [14]. Studies separating vehicle age, crash period, and model year further showed that manufacturing cohort and in-use aging carry distinguishable information about injury outcomes [15,16]. Beyond the overall vehicle-age association, previous research has also examined the role of driver age. In two studies based on Spanish crash data, one reported a more pronounced association with serious injury among older drivers operating older vehicles [17]; the other found that driver age and vehicle age were both associated with injury severity, but their interaction was not statistically significant [18]. Regarding differences by occupants’ own age, a study using data from the National Automotive Sampling System General Estimates System (NASS-GES) and Crash Report Sampling System (CRSS) through 2017 included occupant age in its models but did not directly test whether the association between vehicle age and injury outcomes varied across occupant age groups [19].

Extending the focus from driver age to occupants’ own age also requires linking the vehicle conditions encountered by different populations with their protection needs in crashes. A U.S. population-based study found that younger and older drivers more often drove older vehicles lacking key safety equipment [20]. Vehicles involved in fatal crashes with teenage drivers tend to be older and equipped with fewer safety technologies [21,22]. Related survey evidence further suggests that these differences in vehicle use are shaped by vehicle choice and allocation within households [23]. When the focus is extended from drivers to all crash-involved occupants, age differences also emerge in occupant role and the associated protection environment. Children and adult passengers of different ages differ substantially in seating position, restraint use, and the protection afforded by their seating environment [24,25]. A hospital-based retrospective study in Japan further found that rear-seat occupants had lower odds of in-hospital death and severe trauma than drivers, supporting an association between seating position and injury outcomes [26]. Age differences may also be evident in the associations between crash context and injury outcomes. An age-stratified study of at-fault drivers in South Korea found that associations of the road environment and crash characteristics with injury severity differed across age groups [27]. Beyond these vehicle and contextual factors, human tolerance to crash loads also varies with age. In later life, increasing frailty and declining physiologic reserve raise the probability of serious or fatal injury under similar crash conditions [28,29]. Population-based studies further show that the higher mortality risk among older road users cannot be explained solely by their crash involvement rate [30,31]. At the same time, newer model years are associated with lower fatality risk among older drivers [32,33], further suggesting a relationship between the level of vehicle protection and age-related susceptibility to injury that warrants direct examination. Occupant age therefore reflects not only differences in vehicle use and occupant context, but also differences in the probability of injury after a crash, providing both a theoretical and real-world basis for examining whether vehicle-age associations vary by age.

Age differences in vehicle-age associations are also influenced by effect scale. Odds ratios quantify the relative association between older and newer vehicles on the odds scale, whereas standardized probability differences quantify absolute changes in predicted probability. These measures answer different questions [34,35]. Clearly distinguishing these measures also aligns with the emphasis on interpreting relationships among variables in recent traffic-safety research [36]. When injury probabilities under the reference vehicle-age condition differ across age groups, a group with a lower baseline probability may show a larger relative association. Conversely, a group with a higher baseline probability may have a larger absolute probability change even when its relative association is smaller. A recent study of crash injury severity used average marginal effects to express associations of relevant factors as changes in the predicted probabilities of different injury-severity categories, providing an example of interpreting findings on the probability scale [37]. Although some vehicle-age studies have reported model-predicted probabilities, these analyses have focused on drivers or specific risk combinations [38,39]. In addition, the interpretation of predicted probabilities depends on the underlying covariate distribution. Standardizing predicted probabilities across age groups to a common covariate distribution helps establish a consistent population basis for comparing probabilities and probability differences [34,40]. However, across the full occupant age range, existing research still lacks integrated comparisons of relative associations and standardized probability differences, leaving it unclear whether age-group patterns in vehicle-age associations are consistent across relative and absolute scales.

Against this background, we used nationally representative probability-sample data from the 2016–2024 CRSS [41] to examine the association between vehicle age and fatal or suspected serious injury among occupants involved in U.S. police-reported passenger-vehicle crashes, with particular attention to differences across occupant age groups. Survey-weighted logistic regression accounting for the complex sampling design was used to estimate associations across vehicle-age categories and evaluate age-group heterogeneity. Standardized predicted probabilities and probability differences under a common covariate distribution were further used to compare age patterns on relative and absolute scales. The study addressed the following three questions:

(1) Was increasing vehicle age associated with a graded increase in fatal or suspected serious injury?
(2) Did the relative association between vehicle age and injury outcomes vary across occupant age groups?
(3) Were age-group differences on the relative scale also evident on the absolute scale as reflected by standardized probability differences?

Based on previous evidence, we expected older vehicle age to be associated with worse injury outcomes, with higher odds of fatal or suspected serious injury among occupants of older vehicles than among those of newer vehicles on the relative scale and positive predicted probability differences on the absolute scale. The specific patterns of relative association strength and absolute probability differences across occupant age groups were examined without prespecifying their ordering.

## 2 Materials and methods

The overall study design and analytical workflow are summarized in S1 Fig.

### 2.1 Data source and study population

We conducted a pooled repeated cross-sectional study using the 2016–2024 public-use files from the Crash Report Sampling System (CRSS) of the National Highway Traffic Safety Administration (NHTSA). CRSS uses a stratified, multistage probability sampling design to draw a nationally representative sample of police-reported motor vehicle crashes in the United States. Eligible crashes occur on a trafficway, involve at least one motor vehicle in transport, and result in injury, death, or property damage; approximately 50,000 police crash reports are sampled each year. This study was reported in accordance with the Strengthening the Reporting of Observational Studies in Epidemiology (STROBE) statement [42]. The target population of this study comprised occupants of passenger vehicles involved in U.S. police-reported crashes represented by CRSS.

Person, vehicle, and crash files were linked using crash year and case number, with vehicle number additionally used to link person and vehicle records. Records were included if the CRSS person-type code was 1, 2, or 9 and the involved vehicle was classified under the NHTSA body-type scheme as a passenger car, utility vehicle, van, pickup truck, or other light passenger vehicle. Nonoccupants, motorcycle riders, and occupants of other nonpassenger vehicles, including buses and medium or heavy trucks, were excluded. Records were also required to have a vehicle model year between 1900 and one year after the crash year, an occupant age between 0 and 120 years, and a valid injury-severity code. The primary complete-case analysis was further restricted to records with complete data on occupant sex, seating position, restraint use, vehicle class, U.S. Census region, urbanicity, survey weight, stratum, and primary sampling unit identifiers.

### 2.2 Study variables

Vehicle age was the primary exposure, and police-reported fatal or suspected serious injury was the outcome. Occupant age group was used to assess effect modification, and covariates were used to adjust for differences in the composition of occupants, vehicles, and crash context across vehicle-age groups.

#### 2.2.1 Vehicle age.

Vehicle age was calculated as the crash year minus the vehicle model year. For vehicles with a model year one year later than the crash year, vehicle age was set to 0 years. The primary analysis categorized vehicle age a priori as 0–4, 5–9, 10–14, 15–19, and ≥20 years, with 0–4 years as the reference category.

#### 2.2.2 Fatal or suspected serious injury.

The primary outcome was police-reported fatal or suspected serious injury, defined as K (fatal injury) or A (suspected serious injury) on the KABCO injury-severity scale. Occupants with no apparent injury, possible injury, or suspected minor injury formed the comparison group. Records were excluded if injury severity was unknown or the occupant was coded as having died before the crash.

#### 2.2.3 Occupant age.

Occupant age was categorized a priori into four groups: 0–17, 18–44, 45–64, and ≥65 years, and occupant age group was specified as the primary effect modifier. This grouping was informed by age classifications used in U.S. injury epidemiology research and national injury statistics [43,44]. The 0–17-year group included children, who predominantly travel as passengers, as well as adolescents beginning to assume a driving role, whereas the ≥ 65-year group was used to examine injury outcome patterns among older occupants. The 18–44-year and 45–64-year groups were used to further distinguish adult occupants younger than 65 years [45]. Together, these four age groups were used to assess age-group heterogeneity in the association between vehicle age and fatal or suspected serious injury.

#### 2.2.4 Covariates.

Adjustment variables were selected on the basis of prior literature and a priori subject-matter knowledge [46,47]. Only variables available in every study year that could be harmonized using consistent definitions and coding were retained. The primary models included occupant sex, seating position, restraint use, vehicle class, crash year, U.S. Census region, and urbanicity. Seating position was coded as driver, front passenger, rear passenger, or other. Restraint use was classified as used or not used. Vehicle class included passenger car, utility vehicle, van, pickup truck, and other light passenger vehicle. The reference categories were female for occupant sex, driver for seating position, used for restraint use, and passenger car for vehicle class. Crash year was modeled as a categorical variable, with 2016 as the reference category. The Northeast and urban areas served as the reference categories for U.S. Census region and urbanicity, respectively. Variable definitions, coding, and reference categories for the primary and extended-adjustment models are provided in S1 Table.

Manner of collision, rollover, and extent of vehicle damage were used to characterize the crash process. These variables may lie partly on the pathway between vehicle characteristics and occupant injury. To avoid overadjustment for potential intermediates [48], they were included only as additional covariates in sensitivity analyses.

### 2.3 Statistical analysis

The analyses began with specification of the complex survey design and description of the study sample. Regression models were then used to estimate the overall association between vehicle age and injury outcomes and to test age-group heterogeneity. Standardized predicted probabilities and probability differences were then calculated to compare results across age groups on the absolute scale. Finally, the robustness of the overall vehicle-age association and age-group heterogeneity was evaluated separately under alternative analytic settings. Unless otherwise specified, inference for single-parameter estimates and linear contrasts used a t reference distribution with 42 design degrees of freedom, whereas multiparameter joint hypotheses were evaluated using design-based Wald F tests with 42 denominator degrees of freedom. Prespecified pairwise age-group comparisons in each analysis were adjusted within the corresponding comparison family using the Holm method. All tests were two-sided, with P < 0.05 indicating statistical significance. Statistical analyses were performed using R version 4.6.1 and the survey package version 4.5. Data management was performed using Python version 3.12.13 and pandas version 3.0.1.

#### 2.3.1 Complex survey design.

The survey design was first constructed using all pooled person records. The primary complete-case sample was then specified as a survey domain [49]. The design variables were the final person weight (WEIGHT), stratification variable (PSUSTRAT), and primary sampling unit variable (PSU_VAR). When annual data were pooled, the original CRSS stratum and primary sampling unit identifiers were retained and were not recoded by crash year. Primary variance estimation used a stratified jackknife (JKn) replicate-weight design derived from the original stratified cluster design [50,51]. Variance estimates were centered on the full-sample estimate. The design contained 67 replicate weights and had 42 design degrees of freedom. Taylor series linearization based on the same stratum and primary sampling unit structure was used to verify the variance estimates [52].

The primary analysis sample was described using unweighted counts and survey-weighted percentages. For each vehicle-age category, the survey-weighted proportion of fatal or suspected serious injury was estimated within the primary complete-case survey domain, with 95% confidence intervals (CIs) calculated using JKn replicate-weight variances. Among occupants in vehicles aged ≥20 years, the unweighted median, first and third quartiles, interquartile range, and maximum vehicle age were calculated at the occupant-record level. Variable-specific missingness was also quantified. Survey-weighted distributions of crash year, vehicle age, occupant age, and injury outcome were compared between included and otherwise eligible records excluded for missing covariates.

#### 2.3.2 Regression models.

Regression analyses used survey-weighted logistic regression with a logit link and a quasibinomial variance function. Each model included vehicle-age category, and four models were fitted using a prespecified sequential adjustment scheme. Model 0 adjusted only for crash year. Model 1 additionally included occupant age group, sex, and seating position. Model 2 further added restraint use, vehicle class, U.S. Census region, and urbanicity. Model 3 added all vehicle-age-category × occupant-age-group interaction terms to Model 2. All four models were fitted to the same primary complete-case sample.

Models 0–2 each estimated overall adjusted odds ratios (aORs) comparing each vehicle-age category with vehicles aged 0–4 years, and Model 2 provided the primary estimates of the overall association. Age-group-specific aORs were obtained from linear contrasts of the relevant coefficients in Model 3. Age-group heterogeneity was evaluated using two prespecified joint tests. The 12-degree-of-freedom full interaction test compared the five-category vehicle-age association pattern across the four age groups. The 3-degree-of-freedom core test compared the log(aOR) for vehicles aged ≥20 versus 0–4 years across the four age groups. For the core comparison, all six prespecified pairwise contrasts between age groups were examined, with between-group differences expressed as ratios of odds ratios.

#### 2.3.3 Standardized estimates.

To complement the relative association estimates on the odds-ratio scale, we calculated standardized predicted probabilities for each vehicle-age-category and occupant-age-group combination. For every record in the primary analysis sample, vehicle age and occupant age group were set to the specified combination. Sex, seating position, restraint use, vehicle class, crash year, U.S. Census region, and urbanicity retained their observed values and survey-weighted joint distribution. Individual predicted probabilities were then averaged using survey weights [53,54]. Thus, estimates for all age-group and vehicle-age combinations were standardized to the common survey-weighted covariate distribution of the primary analysis sample.

Within each age group, the standardized probability difference was defined as the standardized predicted probability for vehicles aged ≥20 years minus that for vehicles aged 0–4 years. The delta method was applied using gradients of the standardized estimates with respect to the model coefficients and the full covariance matrix of those coefficients. This approach was used to estimate standard errors for the standardized predicted probabilities and probability differences and to construct the joint covariance matrix of the four probability differences. A 3-degree-of-freedom joint test compared the four age-group-specific probability differences. This joint test, the confidence intervals for the probability differences, and all six pairwise age-group comparisons were based on the same joint covariance matrix.

#### 2.3.4 Robustness analyses of the overall association.

Robustness of the overall vehicle-age association was first evaluated by varying the study period, missing-data handling, vehicle-age range, outcome definition, and sample restriction. For the study period, the analysis was restricted to crashes from 2019–2024. Two separate missing-data analyses were conducted: one used NHTSA single-imputation variables for age, sex, seating position, injury severity, and vehicle model year; the other treated missing values for sex, seating position, and restraint use as separate categories. For the vehicle-age range, vehicles aged >40 years were excluded. For the outcome definition, K (fatal injury) was excluded and A (suspected serious injury) alone was used as the alternative outcome. For the sample restriction, the analysis was restricted to restrained occupants.

Robustness of the overall vehicle-age association was then evaluated with respect to the covariate-adjustment specification. One analysis omitted restraint use from the primary adjustment set, and three separate extended-adjustment models were constructed from Model 2. The driver-characteristics model additionally included the linked driver’s age, sex, alcohol involvement, and speeding-related status. The crash-context model added time of day, lighting conditions, weather, crash location relative to the roadway, and speed limit. The crash-process model added manner of collision, rollover, and extent of vehicle damage. All three extended-adjustment models used Taylor series linearization for variance estimation.

Finally, robustness of the overall vehicle-age association was evaluated across vehicle classes and under alternative modeling of vehicle age. Analyses were stratified by vehicle class, and vehicle-age associations were estimated separately for passenger cars, utility vehicles, vans, and pickup trucks. In the sample restricted to vehicles aged ≤40 years, categorical vehicle age was replaced by a four-degree-of-freedom natural cubic spline for continuous vehicle age. Internal knots were placed at 3, 7, and 13 years, and boundary knots at 0 and 40 years.

#### 2.3.5 Robustness analyses of age-group heterogeneity.

Robustness of age-group heterogeneity was first assessed by varying the study period, missing-data handling, vehicle-age range, sample restriction, and age grouping. The study-period analysis was restricted to crashes from 2019–2024, whereas the missing-data analysis used NHTSA single-imputation variables, as described in Section 2.3.4. For the vehicle-age range, analyses were restricted to occupants of vehicles aged ≤40 years. For sample restrictions, separate analyses were conducted among restrained occupants, drivers, and adult occupants (≥18 years). For age grouping, occupants were classified as minors (0–17 years) or adults (≥18 years). Among occupants aged 0–17 years, age was further categorized as 0–8, 9–15, or 16–17 years to test vehicle-age-category × age-subgroup interactions under alternative groupings. These analyses used Taylor series linearization for variance estimation, with the minor-versus-adult analysis additionally verified using JKn replicate weights.

To examine age-related variation in vehicle-age associations without relying on the prespecified age-group boundaries, occupant age was also modeled continuously. In the primary complete-case sample, continuous occupant age was represented by a three-degree-of-freedom natural cubic spline and entered into a full interaction model with the five-category vehicle-age variable. This model replaced the four-category age group with the age spline, retained the other covariates from Model 2, and used JKn replicate weights for variance estimation. A 12-degree-of-freedom joint test assessed all vehicle-age-category × age-spline interaction terms. For ages 0–100 years, aORs comparing vehicles aged ≥20 with those aged 0–4 years were estimated from linear combinations of model coefficients. Pointwise 95% confidence intervals were calculated using the full coefficient covariance matrix. The age spline was further increased to four degrees of freedom, and the full interaction was assessed using a 16-degree-of-freedom joint test.

Age-group heterogeneity was further examined with respect to occupant role and within-group age composition. The role analysis collapsed seating position into driver and passenger categories and fitted a vehicle-age-category × occupant-role interaction model. Because occupant role was derived from seating position, the four-category seating-position variable was omitted from this model. Vehicle-age-category × occupant-age-group models were then fitted separately among drivers and passengers. The passenger model adjusted for front, rear, and other seating positions. In the within-group continuous-age analysis, continuous age was centered at the survey-weighted mean within each age group. Age-group-specific within-group continuous-age terms were then added to Model 3 without interactions between vehicle age and continuous age.

Finally, the sensitivity of age-group heterogeneity to outcome composition was examined. The fatal-outcome analysis used K (fatal injury) as the outcome and fitted Models 2 and 3. Except for the seating-position handling required by the occupant-role definition, the occupant-role, within-group continuous-age, and fatal-outcome analyses used the survey design, covariates, and inference methods of the primary analysis.

### 2.4 Ethics statement

The CRSS public-use files were accessed for research purposes on 25 July 2026. These files are publicly available and deidentified. The authors did not participate in primary data collection and had no access at any time to information that could identify individual crash-involved persons. This study involved no prospective recruitment or direct participant contact. Because it was a secondary analysis of publicly available, deidentified data, ethics committee approval and informed consent were not required.

## 3 Results

### 3.1 Study sample overview

Of 1,158,730 person records, 1,015,530 met the passenger-vehicle and occupant-type definitions. Among these records, 951,314 had valid vehicle age, occupant age, and injury outcome data, and 899,273 were included in the primary complete-case analysis. The primary analysis sample represented 94.5% of these 951,314 records and 94.6% of their corresponding total survey weight. The main sources of missing data were restraint use (weighted missingness, 4.79%), seating position (0.46%), and sex (0.31%); missingness could overlap across variables. Among otherwise eligible records excluded because of missing covariate data, the weighted proportion of fatal or suspected serious injury was 1.93%, compared with 1.08% among included records. The weighted proportion of records involving vehicles aged ≥20 years was 8.48% among excluded records and 6.27% among included records (S2 Table).

In the primary complete-case sample, 53,218 occupant records involved vehicles aged ≥20 years. At the occupant-record level, vehicle age had an unweighted median of 22 years, first and third quartiles of 21 and 25 years, an interquartile range of 4 years, and a maximum of 100 years. The primary analysis sample included 46,367 occupants with fatal or suspected serious injury. After survey weighting, occupants aged 0–17, 18–44, 45–64, and ≥65 years accounted for 13.3%, 53.8%, 22.9%, and 10.1% of the sample, respectively; 47.8% were female. Drivers, front passengers, and rear passengers accounted for 75.3%, 14.2%, and 10.5%, respectively, and 98.0% of occupants used restraints. Passenger cars and utility vehicles accounted for 53.1% and 25.4% of the sample. Pickup trucks, vans, and other light passenger vehicles accounted for 12.9%, 5.5%, and 3.1%, respectively. Overall, 77.4% of occupants were involved in urban crashes.

Occupant and vehicle composition varied across vehicle-age categories. The weighted proportion of male occupants was 48.4% in vehicles aged 0–4 years and 69.1% in those aged ≥20 years. The corresponding weighted proportions were 1.3% and 3.9% for restraint nonuse, 10.6% and 30.2% for occupants in pickup trucks, and 19.1% and 30.5% for occupants involved in rural crashes (S3 Table). Seating-position composition also varied by occupant age group. Among occupants aged 0–17 years, 56.9% were rear passengers, whereas 82.2%–86.2% of occupants in the three adult age groups were drivers (S4 Table). All 20 age-group × vehicle-age-category cells contained at least 6,169 occupant records and 442 outcome events. Unweighted sample sizes, event counts, and survey-weighted outcome proportions for each cell are reported in S5 Table.

### 3.2 Overall vehicle-age association

The survey-weighted proportion of fatal or suspected serious injury increased across vehicle-age categories, from 0.72% (95% CI, 0.64%–0.80%) in vehicles aged 0–4 years to 2.15% (1.93%–2.36%) in those aged ≥20 years (S6 Table). Across Models 0–2, the aOR point estimates increased with vehicle age. Compared with Model 1, the aORs in Model 2 were attenuated for all four nonreference vehicle-age categories, although the graded pattern remained. In Model 2, the aOR comparing vehicles aged ≥20 years with those aged 0–4 years was 2.34 (95% CI, 2.19–2.49) (Table 1).

**Table 1:** Sequentially adjusted odds ratios for fatal or suspected serious injury by vehicle age (Models 0–2).

Model	0–4 years	5–9 years	10–14 years	15–19 years	≥20 years
M0	1.00 (reference)	1.20 (1.14–1.26)	1.67 (1.59–1.75)	2.28 (2.15–2.43)	3.05 (2.86–3.26)
M1	1.00 (reference)	1.21 (1.16–1.27)	1.70 (1.63–1.78)	2.33 (2.20–2.46)	3.06 (2.87–3.26)
M2	1.00 (reference)	1.14 (1.09–1.19)	1.50 (1.43–1.57)	1.94 (1.84–2.05)	2.34 (2.19–2.49)

Note. Values are adjusted odds ratios (aORs) with 95% confidence intervals; vehicles aged 0–4 years are the reference. Model definitions are provided in Section 2.3.2.

### 3.3 Age heterogeneity on the relative scale

In Model 3, the full vehicle-age-category × occupant-age-group interaction was statistically significant [F(12, 42) = 2.17, P = 0.032]. In the prespecified core comparison, the log(aOR) for vehicles aged ≥20 versus 0–4 years also differed across the four age groups [F(3, 42) = 3.64, P = 0.020]. For vehicles aged ≥20 versus 0–4 years, the aOR was 2.80 (95% CI, 2.27–3.44) among occupants aged 0–17 years and 2.39 (2.23–2.57) among those aged 18–44 years. The corresponding aORs were 2.31 (2.07–2.57) among occupants aged 45–64 years and 1.99 (1.75–2.26) among those aged ≥65 years (Table 2; Fig 1).

**Table 2:** Age-group-specific adjusted odds ratios for the association between vehicle age and fatal or suspected serious injury (Model 3).

Vehicle age (years)	0–17 years	18–44 years	45–64 years	≥65 years
0–4	1.00 (reference)	1.00 (reference)	1.00 (reference)	1.00 (reference)
5–9	1.15 (0.99–1.32)	1.14 (1.07–1.23)	1.11 (1.03–1.19)	1.18 (1.07–1.30)
10–14	1.64 (1.40–1.91)	1.51 (1.41–1.63)	1.51 (1.40–1.62)	1.38 (1.20–1.58)
15–19	2.19 (1.85–2.60)	1.96 (1.82–2.11)	2.02 (1.86–2.20)	1.65 (1.44–1.88)
≥20	2.80 (2.27–3.44)	2.39 (2.23–2.57)	2.31 (2.07–2.57)	1.99 (1.75–2.26)

Note. Values are age-group-specific adjusted odds ratios (aORs) with 95% confidence intervals (CIs). Within each age group, vehicles aged 0–4 years served as the reference category; model definitions are provided in Section 2.3.2.

**Fig 1:**
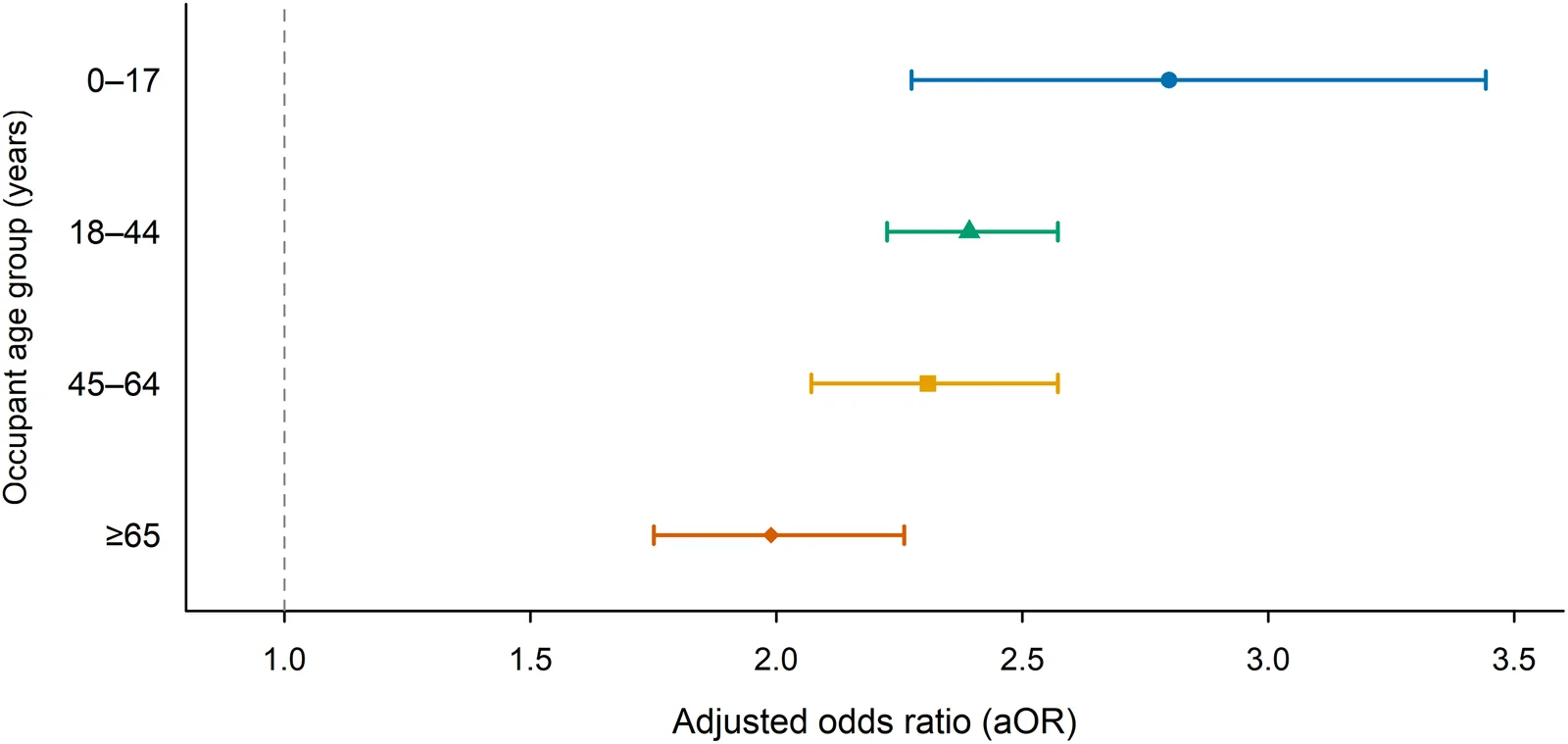
Adjusted odds ratios comparing vehicles aged ≥20 years with those aged 0–4 years across occupant age groups. Note. Points represent age-group-specific aORs estimated from Model 3, and error bars represent 95% confidence intervals based on the t distribution with 42 design degrees of freedom. Within each age group, vehicles aged 0–4 years served as the reference category.

For occupants aged 0–17 versus ≥65 years, the ratio of odds ratios was 1.41 (95% CI, 1.11–1.78; Holm-adjusted P = 0.035). The corresponding ratio for occupants aged 18–44 versus ≥65 years was 1.20 (95% CI, 1.05–1.38; Holm-adjusted P = 0.047). The remaining four pairwise age-group comparisons were not statistically significant (S7 Table). Taylor series linearization yielded results closely consistent with those obtained using JKn replicate weights for both the full vehicle-age-category × occupant-age-group interaction [F(12, 42) = 2.17, P = 0.032] and the core comparison between vehicles aged ≥20 and 0–4 years [F(3, 42) = 3.67, P = 0.019] (S8 Table).

### 3.4 Age heterogeneity on the absolute scale

After standardization to the common survey-weighted covariate distribution of the primary analysis sample, occupants aged ≥65 years had the highest standardized predicted probability in every vehicle-age category. The standardized predicted probability for this group was 1.23% (95% CI, 1.09%–1.38%) for vehicles aged 0–4 years and 2.36% (2.01%–2.70%) for those aged ≥20 years. Standardized predicted probabilities for all age-group and vehicle-age combinations and their confidence intervals are shown in Fig 2A and S9 Table, panel A.

**Fig 2:**
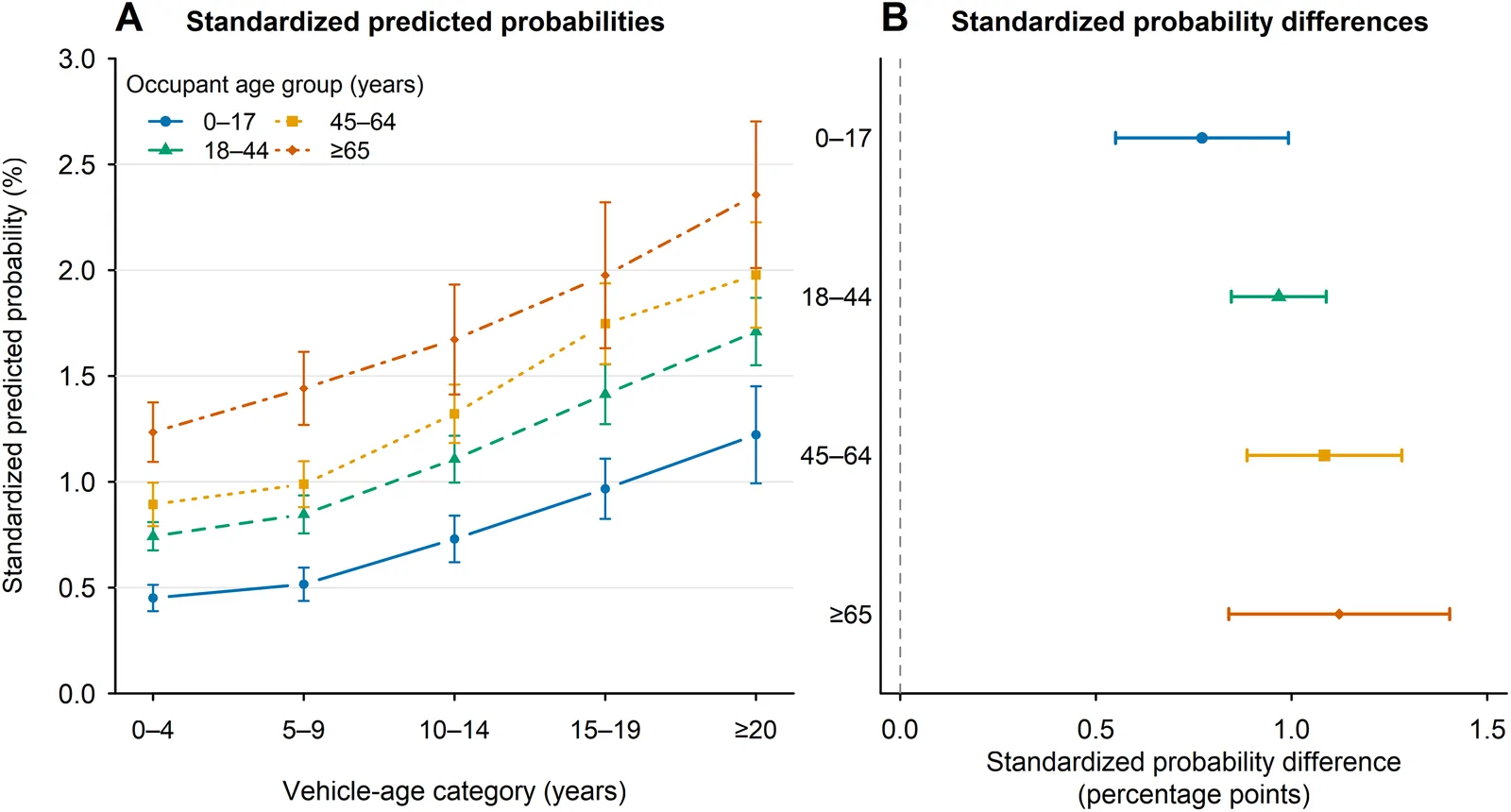
Standardized predicted probabilities and probability differences for vehicle age and fatal or suspected serious injury across occupant age groups. Note. Panel A shows standardized predicted probabilities for each age group across the five vehicle-age categories. Panel B shows standardized probability differences, expressed in percentage points, for vehicles aged ≥20 years minus those aged 0–4 years. All estimates were derived from Model 3 and standardized to the same survey-weighted joint covariate distribution of the primary analysis sample. Points represent estimates, and error bars represent 95% confidence intervals based on the t distribution with 42 design degrees of freedom.

For vehicles aged ≥20 versus 0–4 years, the standardized probability difference was 0.77 percentage points (95% CI, 0.55–0.99) among occupants aged 0–17 years and 0.97 percentage points (0.85–1.09) among those aged 18–44 years. The corresponding probability differences were 1.08 percentage points (0.89–1.28) among occupants aged 45–64 years and 1.12 percentage points (0.84–1.40) among those aged ≥65 years. The point estimates of the standardized probability differences increased from 0.77 to 1.12 percentage points across the four age groups, but the overall heterogeneity test was not statistically significant [F(3, 42) = 2.41, P = 0.080]. After Holm adjustment, none of the six pairwise comparisons across age groups was statistically significant, with all adjusted P values ≥0.138 (Fig 2B; S9 Table, panels B–C).

### 3.5 Robustness of the overall association

The overall aOR comparing vehicles aged ≥20 with those aged 0–4 years remained close to the primary estimate after varying the study period, missing-data handling, vehicle-age range, outcome definition, and analysis sample. The aOR was 2.29 (95% CI, 2.13–2.46) when the analysis was restricted to 2019–2024 crashes and 2.31 (2.17–2.46) when NHTSA single-imputation variables were used. It was 2.32 (2.19–2.47) when missing sex, seating position, and restraint use were represented as separate categories, and 2.33 (2.19–2.48) after vehicles aged >40 years were excluded. The aOR was 2.29 (2.14–2.45) when A (suspected serious injury) alone was used as the outcome and 2.33 (2.17–2.50) among restrained occupants. These sensitivity analyses are summarized in S2 Fig, with complete estimates in S10 Table, panel A.

The overall association comparing vehicles aged ≥20 with those aged 0–4 years also remained evident after changes to the covariate-adjustment specification. After restraint use was omitted from the primary adjustment set, the aOR comparing vehicles aged ≥20 with those aged 0–4 years was 2.88 (95% CI, 2.69–3.09). After records without a linked driver were excluded, the driver-characteristics extended model included 899,091 occupant records and 46,349 K/A events, and the aOR was 2.17 (2.03–2.32). The corresponding aORs for the crash-context and crash-process extended models were 2.09 (1.99–2.20) and 1.60 (1.50–1.69), respectively (S10 Table, panel B).

The overall vehicle-age association was also observed in vehicle-class-stratified and continuous vehicle-age analyses. The direction of association was consistent across the four vehicle classes examined. The aOR comparing vehicles aged ≥20 with those aged 0–4 years was 2.11 (95% CI, 1.97–2.26) for passenger cars and 2.86 (2.52–3.24) for utility vehicles. The corresponding estimates were 2.46 (1.88–3.22) for vans and 2.44 (2.17–2.75) for pickup trucks (S10 Table, panel C). Among vehicles aged ≤40 years, the overall association between continuous vehicle age, represented by a natural cubic spline, and fatal or suspected serious injury was statistically significant [F(4, 42) = 297.51, P < 0.001]. Representative estimates are reported in S11 Table.

### 3.6 Robustness of age heterogeneity

The statistical evidence for the full interaction varied across sensitivity analyses involving the study period, missing-data handling, vehicle-age range, sample restrictions, and age grouping. The full vehicle-age-category × occupant-age-group interaction remained statistically significant in the 2019–2024 crash sample, the sample using NHTSA single-imputation variables, and restrained occupants. It also remained statistically significant among occupants of vehicles aged ≤40 years. When the analysis was restricted to adult occupants aged ≥18 years, the interaction test was not statistically significant (P = 0.095). When age was regrouped as minors and adults, the interaction-test P values were 0.140 with JKn replicate weights and 0.141 with Taylor series linearization. Occupants aged 0–17 years were further categorized as 0–8, 9–15, and 16–17 years. The resulting vehicle-age-category × age-subgroup interaction test yielded P = 0.107. Complete results are reported in S12 Table.

When occupant age was modeled using a three-degree-of-freedom natural cubic spline, the full vehicle-age-category × age-spline interaction was statistically significant [F(12, 42) = 3.41, P = 0.002]. A model using a four-degree-of-freedom age spline also yielded a statistically significant full interaction [F(16, 42) = 2.84, P = 0.003]. For the comparison between vehicles aged ≥20 and 0–4 years, the aOR curves from both models varied continuously across age, with local fluctuations during young and middle adulthood and an overall attenuation at older ages. Results at representative ages and sample support across age bands are reported in S13 Table, and the age-specific curves are shown in S3 Fig.

### 3.7 Supplementary analyses of heterogeneity

In the occupant-role analysis, there were 662,454 drivers and 236,819 passengers, with 35,063 and 11,304 fatal or suspected serious injury events, respectively. The vehicle-age-category × occupant-role interaction test yielded F(4, 42) = 2.29 and P = 0.076. For vehicles aged ≥20 versus 0–4 years, the aOR was 2.66 (95% CI, 2.40–2.96) among passengers and 2.27 (2.11–2.44) among drivers; the between-role difference in this comparison was statistically significant [F(1, 42) = 7.68, P = 0.008]. After stratification by occupant role, the P values for the full vehicle-age-category × occupant-age-group interaction were 0.081 among drivers and 0.072 among passengers. The corresponding P values for the core age-group heterogeneity test were 0.196 and 0.287, respectively (S14 Table).

In the within-group age-composition analysis, after age-group-specific continuous-age terms were added to Model 3, the full vehicle-age-category × occupant-age-group interaction was not statistically significant [F(12, 42) = 1.78, P = 0.083]. The core age-group heterogeneity test for vehicles aged ≥20 versus 0–4 years remained statistically significant [F(3, 42) = 3.48, P = 0.024]. The corresponding aORs were 2.58 (95% CI, 2.09–3.19) among occupants aged 0–17 years and 2.43 (2.26–2.61) among those aged 18–44 years. They were 2.31 (2.07–2.57) among occupants aged 45–64 years and 1.97 (1.74–2.24) among those aged ≥65 years. After Holm adjustment, only the comparison between occupants aged 18–44 and ≥65 years was statistically significant (adjusted P = 0.021) (S15 Table).

The analysis restricted to fatal injury (K) included 6,270 events. The overall vehicle-age association retained a graded pattern, with an aOR of 2.49 (95% CI, 2.27–2.73) for vehicles aged ≥20 versus 0–4 years. The full vehicle-age-category × occupant-age-group interaction yielded F(12, 42) = 2.83 and P = 0.006. The age-group heterogeneity test for vehicles aged ≥20 versus 0–4 years yielded F(3, 42) = 0.91 and P = 0.446. None of the six pairwise age-group comparisons was statistically significant after Holm adjustment. Complete results are reported in S16 Table.

## 4 Discussion

### 4.1 Real-world interpretation of the vehicle-age gradient

This study observed a graded increase in the odds of fatal or suspected serious injury with vehicle age among occupants involved in police-reported crashes, and this association persisted across multiple sensitivity analyses. Similar vehicle-age associations have been reported previously [19,55]. By extending the CRSS data through 2024, the present study further shows that the overall gradient remained consistent across alternative analytic settings and vehicle classes. Thus, the association between vehicle age and serious injury is not limited to earlier crash data but remains evident in the contemporary U.S. crash-involved fleet. This persistent association suggests that the real-world significance of vehicle age should be considered in terms of both the temporal characteristics of the vehicle itself and the context in which vehicles are distributed and used.

The vehicle-age gradient remained after adjustment for crash year, and continuous vehicle-age modeling also supported the overall association, suggesting that vehicle age captures safety-related differences beyond temporal variation across crash years. At the time of manufacture, vehicles reflect the structural design, occupant-protection technologies, and regulatory standards of a particular period [56]. After entering service, their condition may continue to change through prolonged use, maintenance, and repair. Previous research has also shown that, even among vehicles no more than three years old, the proportion of serious injury declined between 2010 and 2022 [57], suggesting that similar time in use does not necessarily imply equivalent levels of crash protection. In this context, vehicle age is better understood not simply as the number of years a vehicle has been in service, but as an indicator of broader safety differences between newer and older vehicles in the real-world fleet, reflecting both technological generation and condition changes accumulated during use.

Beyond technological generation and in-use history, vehicle age is also related to how vehicles are distributed and used within the transportation system. Older vehicles in this study were more often pickup trucks and were more frequently involved in rural crashes or crashes involving unrestrained occupants. The vehicle-age association attenuated to varying degrees when driver characteristics, crash context, and crash-process factors were considered in separate models. An Ohio crash study similarly found that earlier model years co-occurred with restraint nonuse, roadway departure, and speed-related factors in combinations associated with serious or fatal injury [39]. Broader traffic-safety research has likewise shown that fatal-crash frequency is associated with traffic environments and broader contextual conditions [58]. Taken together, these findings suggest that vehicles of different ages are not distributed uniformly across the same populations and traffic environments, and that vehicle-age associations occur alongside differences in how vehicles are used and the crash contexts in which they are involved. Interpretation of the vehicle-age gradient therefore requires consideration of both vehicle-related temporal characteristics and the broader context of vehicle use.

### 4.2 Age-group differences in vehicle-age associations

The primary analysis showed that vehicle-age associations differed across occupant age groups. Both the full five-category vehicle-age pattern and the core comparison between vehicles aged ≥20 and 0–4 years provided statistical evidence of age-group heterogeneity. In the core comparison, the aOR point estimate was highest among occupants aged 0–17 years, generally declined across older age groups, and was lowest among those aged ≥65 years. This finding extends previous age-comparison research focused mainly on drivers [17,18] to the full occupant population, including children, adult passengers, and drivers. Because age groups also differ in life stage, occupant role, and seating composition, these characteristics provide important context for interpreting the age pattern observed in the primary analysis.

Robustness analyses using alternative representations of age further clarified the form of these differences. Neither dichotomizing age into minors and adults nor further subdividing occupants aged 0–17 years produced a statistically significant joint interaction test. In contrast, the full vehicle-age × age interaction was statistically significant when continuous age was modeled using natural cubic splines with three or four degrees of freedom. The corresponding aOR curve comparing vehicles aged ≥20 with those aged 0–4 years varied continuously across age, with local fluctuations during young and middle adulthood and an overall attenuation at older ages. Different representations of age therefore yielded different patterns of statistical evidence. The continuous-age models also characterized variation across age beyond the four prespecified groups.

The occupant-role analysis further refined the interpretation of this heterogeneity. In the core comparison, the relative association was stronger among passengers than among drivers, whereas the occupant-role interaction across the full vehicle-age pattern was not statistically significant. When drivers and passengers were analyzed separately, neither the full nor the core age-group interaction test reached statistical significance in either subgroup. This finding was consistent with the sample composition. More than half of occupants aged 0–17 years were rear passengers, whereas the adult age groups consisted predominantly of drivers. This pattern indicates substantial differences in occupant role and seating composition across age groups. Injury outcomes among child occupants are also associated with seating position, restraint use, and vehicle characteristics [59,60].

After further adjustment for within-group continuous age while retaining the four age groups, statistical evidence of age-group differences across the full vehicle-age pattern was attenuated, whereas heterogeneity in the core comparison remained. When the outcome was restricted to fatal injury, age-group differences were evident across the full five-category vehicle-age pattern but not in the ≥ 20-versus-0–4-year comparison. Taken together, these supplementary analyses distinguish several manifestations of age heterogeneity. Occupant-role differences were most apparent in the core comparison, while within-group age composition affected the evidence across the full vehicle-age pattern. The fatal-injury analysis showed a different distribution of heterogeneity across vehicle-age contrasts. Age group in this study therefore also captures differences in life stage, occupant role, and occupant-protection environment. Recent injury-severity research has similarly emphasized heterogeneity in driver, vehicle, and crash-context characteristics when explaining variation in injury outcomes [61].

### 4.3 Age-group differences on relative and absolute scales

Age-group patterns differed across three measures: relative association strength, standardized predicted probability, and standardized probability difference. In the comparison between vehicles aged ≥20 and 0–4 years, occupants aged 0–17 years had the highest aOR point estimate, whereas those aged ≥65 years had the lowest. Across all vehicle-age categories, however, occupants aged ≥65 years consistently had the highest standardized predicted probabilities. Thus, the ordering of age groups differed between relative association strength and absolute injury probability. Younger occupants showed a more pronounced relative association when older and newer vehicles were compared, whereas older occupants maintained higher standardized predicted probabilities across vehicle-age conditions. This pattern is consistent with the greater susceptibility to injury associated with reduced physical tolerance and diminished physiological reserve in later life [30]. Point estimates of standardized probability differences also increased across age groups, from 0.77 percentage points among occupants aged 0–17 years to 1.12 percentage points among those aged ≥65 years. However, neither the overall heterogeneity test nor the Holm-adjusted pairwise comparisons reached statistical significance.

These differences arise because the effect measures address different statistical questions. The aOR describes the covariate-conditional relative association in outcome odds between older and newer vehicles. Standardized predicted probabilities represent marginal injury probabilities under a specified vehicle-age condition after averaging over a common covariate distribution, whereas standardized probability differences quantify the absolute change between two vehicle-age categories. Absolute probability differences depend on both the relative magnitude of the vehicle-age association and the baseline probability; consequently, a larger aOR does not necessarily correspond to a larger probability difference. Standardizing all age groups to this common distribution places the absolute measures on a common population basis and improves comparability across age groups.

Methodological research has shown that subgroup differences for binary outcomes may vary depending on whether effects are evaluated on relative or absolute scales, and that reporting both scales provides a more complete description of population heterogeneity [62,63]. In the present study, age-group-specific aORs, standardized predicted probabilities, and standardized probability differences were incorporated into the same analytic framework. These measures characterized three distinct features: the relative association between older and newer vehicles, the injury probability under a given vehicle-age condition, and the absolute probability change between vehicle-age categories. These measures answer different questions rather than representing alternative numerical expressions of the same association. Together, they show that the interpretation of age-group differences depends on the statistical scale under consideration.

### 4.4 Practical implications for vehicle safety

The findings of this study suggest that vehicle age can serve as a simple starting point for stratifying vehicle safety within the real-world fleet. As the preceding analyses suggest, vehicle age may reflect differences in vehicle technology generation, in-use history, and real-world use context. Building on vehicle-age stratification, the occupant-protection performance of individual vehicles can be further assessed using crash safety ratings, key safety features, recall completion, and maintenance records, allowing additional evaluation to focus on vehicles that may differ substantially in protection performance. Household vehicle allocation and the used-vehicle market are the most direct settings for applying this approach. When household resources constrain access to vehicles with higher levels of protection, the availability of safer vehicles also influences how safety technologies are distributed across the real-world fleet [64]. The practical value of vehicle age therefore lies in translating a population-level vehicle-age gradient into a basis for further assessment of individual vehicles and in providing more targeted safety information for vehicle choice and used-vehicle transactions.

At the fleet-governance level, vehicle inspection is an important component of efforts to improve vehicle condition and safety performance. A systematic review and a Danish nationwide registry study found no consistent reduction in crashes from increasing inspection frequency alone [65,66], suggesting that inspection programs are most useful when they identify safety-critical defects, lead to completed repairs, and improve vehicle condition. Previous traffic-safety policy research has also shown that the effects of regulatory measures on fatal-crash frequency may vary over time and require longitudinal data and dedicated policy-evaluation designs for evaluation [67]. Vehicle safety governance can therefore be oriented toward improving actual occupant-protection performance, while fleet renewal can promote the broader diffusion of established safety technologies across the in-use fleet. In this sense, fleet renewal links generational replacement of vehicles with the translation of advances in vehicle safety into population-level safety benefits.

### 4.5 Limitations and future directions

This study has several limitations. The CRSS study population is restricted to occupants involved in police-reported crashes, so inclusion is conditioned on both crash occurrence and police reporting. In such a crash-conditioned sample, selection bias or collider bias may arise when vehicle age and related factors also influence crash involvement. Accordingly, the estimates primarily describe associations with injury severity after a crash, whereas crash-involvement risk requires investigation using broader exposure data. Across CRSS years, consistent measures of vehicle safety status and crash processes were unavailable. K/A classifications were derived from police records and have a different measurement basis from clinical trauma assessments, which may contribute to residual confounding and measurement error. In addition, older vehicles and severe-injury outcomes were more common among records excluded from the complete-case analysis, while sparse data at extreme occupant ages, very high vehicle ages, and in some stratified analyses resulted in limited empirical overlap across vehicle-age categories, representing a potential positivity limitation. The primary comparisons were evaluated using prespecified tests with adjustment for multiple comparisons, but the larger number of supplementary analyses still increases the possibility of chance findings. Continuous-age models reduced reliance on prespecified age categories, but the fixed-parameter models used here primarily characterize average differences and do not directly identify unobserved individual heterogeneity.

Future research can move beyond vehicle age as a composite marker toward more specific measures of vehicle safety status. Linking crash records to vehicle identification number (VIN)-level information on safety features, crash ratings, recall completion, and maintenance could help distinguish the contributions of vehicle technology generation and condition accumulated through long-term use. Further integration of crash reconstruction, event data recorder outputs, and clinical trauma data could trace the links among crash loads, occupant protection, and resulting injury. Linkage with vehicle registration and mileage data could extend research from post-crash injury severity to crash involvement and exposure risk, while also supporting evaluation of vehicle inspection, recall, repair, and fleet-renewal measures. Methodologically, random-parameter and related models could be used to investigate individual-level heterogeneity not captured by the current analyses.

## 5 Conclusions

In nationally representative CRSS data from 2016–2024, vehicle age was consistently associated with a graded increase in fatal or suspected serious injury among occupants involved in police-reported crashes, and this pattern was robust across multiple analytic settings. Age-group differences were most evident on the relative scale. In the comparison between vehicles aged ≥20 and 0–4 years, occupants aged 0–17 years had the highest aOR point estimate, whereas those aged ≥65 years had the lowest; continuous-age analysis further showed that the relative association generally attenuated with increasing age. After standardization to a common covariate distribution, occupants aged ≥65 years had the highest standardized predicted probability in every vehicle-age category, whereas the overall comparison of standardized probability differences across age groups did not reach statistical significance. Thus, the relative strength of the vehicle-age association, injury probability under a given vehicle-age condition, and absolute probability change between older and newer vehicles reflected different population patterns. Considering these measures together provides a more complete understanding of vehicle-age differences in the real-world fleet and provides a basis for vehicle safety stratification and subsequent safety assessment.

## Supporting information

S1 Fig Schematic overview of the study framework. The figure outlines the overall approach of this study.
(ZIP)

S2 Fig Sensitivity analyses comparing vehicles aged ≥20 years with those aged 0–4 years. Points and horizontal lines show adjusted odds ratios and 95% confidence intervals on a logarithmic horizontal axis. The three sections correspond to alternative analysis settings in S10 Table Panel A, alternative covariate adjustment sets in Panel B, and vehicle-class-specific analyses in Panel C. Numerical estimates, sample sizes, and analysis-specific variance and confidence-interval conventions are reported in S10 Table.
(ZIP)

S3 Fig Continuous-age pattern of the association between vehicles aged ≥20 versus 0–4 years and fatal or suspected serious injury. The solid line shows age-specific adjusted odds ratios (aORs) from the primary continuous occupant-age natural cubic spline interaction model (df = 3). Shading shows pointwise 95% confidence intervals calculated using JKn variance and t(42), and the dotted line denotes aOR=1. The vertical axis is logarithmic, and the curve is displayed for ages 0–100 years. Model specifications, joint tests, representative-age estimates, and age-band sample support are reported in S13 Table.
(ZIP)

S1 Table Variable coding and reference categories for the primary and extended models. Source and analysis variables, coding, reference categories, missing-data handling, and model-specific sample exclusions are reported.
(DOCX)

S2 Table Missingness and comparison of included and excluded eligible records. Panel A reports sample-selection stages and missing primary covariates; Panel B compares included and excluded eligible records.
(DOCX)

S3 Table Characteristics of the primary analysis sample by vehicle age, CRSS 2016–2024. Sample characteristics are reported overall and within each vehicle-age category.
(DOCX)

S4 Table Seating-position composition within occupant age groups. Counts and weighted seating-position distributions are reported for each occupant age group.
(DOCX)

S5 Table Sample support and injury proportions within occupant-age-by-vehicle-age cells. Sample sizes, K/A event counts, weighted outcome proportions, and 95% confidence intervals are reported for all 20 cells.
(DOCX)

S6 Table Survey-weighted proportion of fatal or suspected serious injury by vehicle-age category. Sample sizes, K/A event counts, weighted outcome proportions, and 95% confidence intervals are reported for the five vehicle-age categories.
(DOCX)

S7 Table Pairwise comparisons of age-specific adjusted odds ratios for vehicles aged ≥20 versus 0–4 years. Six age-group comparisons report aOR ratios, 95% confidence intervals, raw P values, and Holm-adjusted P values.
(DOCX)

S8 Table Comparison of JKn replicate-weight and Taylor series linearization estimates. Panel A compares core joint tests; Panel B compares core effect estimates and standard-error ratios.
(DOCX)

S9 Table Standardized predicted probabilities, probability differences, and age-group comparisons. Panel A reports standardized predicted probabilities; Panel B reports ≥20-minus-0–4-year probability differences. Panel C reports their joint age-group equality test and six Holm-adjusted pairwise comparisons.
(DOCX)

S10 Table Sensitivity analyses of the overall association comparing vehicles aged ≥20 with those aged 0–4 years. Panel A evaluates robustness to alternative analysis settings; Panel B presents alternative covariate adjustment sets; Panel C presents vehicle-class-specific analyses.
(DOCX)

S11 Table Continuous vehicle-age spline estimates relative to vehicle age 0 years. Representative aORs, 95% confidence intervals, and P values are reported.
(DOCX)

S12 Table Robustness analyses of age-group heterogeneity. Interaction tests evaluate alternative analysis settings, sample restrictions, and alternative age groupings.
(DOCX)

S13 Table Continuous occupant-age spline models, representative estimates, and sample support. Panel A reports specifications and joint tests for the 3-df and 4-df models. Panel B gives representative-age estimates. Panel C reports sample support by five-year age band.
(DOCX)

S14 Table Vehicle-age associations and age heterogeneity by occupant role. Panel A compares vehicle-age associations between drivers and passengers. Panel B reports age heterogeneity within driver and passenger strata.
(DOCX)

S15 Table Age-group heterogeneity after within-group continuous-age adjustment. Age-group-specific aORs, joint tests, and pairwise comparisons are reported after within-group continuous-age adjustment.
(DOCX)

S16 Table Vehicle-age associations and age heterogeneity for fatal injury. Overall and age-group-specific vehicle-age associations, joint tests, and pairwise comparisons are reported for K-level fatal injury.
(DOCX)

S1 File Reproducibility package for the CRSS vehicle-age and occupant-age analyses. The package contains the analysis dataset, data documentation, code for reproducing the reported analyses, and software-environment information.
(ZIP)
