# Supplementary material for: Do older vehicles affect occupants of all ages equally? Age-group differences in the association between vehicle age and fatal or suspected serious injury in U.S. police-reported passenger-vehicle crashes, 2016–2024
Source: PLoS One. 2026 Nov 12;21(11):e0362075. doi: 10.1371/journal.pone.0362075 (PMC13759116; doi:10.1371/journal.pone.0362075)
Supplement: Supplementary file 1 [file pone.0362075.s001.zip › S1_Fig/S1_Fig.docx]

**S1 Fig. Schematic overview of the study framework.**
