# Supplementary material for: Do older vehicles affect occupants of all ages equally? Age-group differences in the association between vehicle age and fatal or suspected serious injury in U.S. police-reported passenger-vehicle crashes, 2016–2024
Source: PLoS One. 2026 Nov 12;21(11):e0362075. doi: 10.1371/journal.pone.0362075 (PMC13759116; doi:10.1371/journal.pone.0362075)
Supplement: Supplementary file 2 [file pone.0362075.s002.zip › S2_Fig/S2_Fig.docx]

**S2 Fig. Sensitivity analyses comparing vehicles aged ≥20 years with those aged 0–4 years.**

Note. Points and horizontal lines show adjusted odds ratios and 95% confidence intervals on a logarithmic horizontal axis. The three sections correspond to alternative analysis settings in S10 Table Panel A, alternative covariate adjustment sets in Panel B, and vehicle-class-specific analyses in Panel C. Numerical estimates, sample sizes, and analysis-specific variance and confidence-interval conventions are reported in S10 Table.
