# Supplementary material for: Do older vehicles affect occupants of all ages equally? Age-group differences in the association between vehicle age and fatal or suspected serious injury in U.S. police-reported passenger-vehicle crashes, 2016–2024
Source: PLoS One. 2026 Nov 12;21(11):e0362075. doi: 10.1371/journal.pone.0362075 (PMC13759116; doi:10.1371/journal.pone.0362075)
Supplement: Supplementary file 3 [file pone.0362075.s003.zip › S3_Fig/S3_Fig.docx]

**S3 Fig. Continuous-age pattern of the association between vehicles aged ≥20 versus 0–4 years and fatal or suspected serious injury.**

Note. The solid line shows age-specific adjusted odds ratios (aORs) from the primary continuous occupant-age natural cubic spline interaction model (df=3). Shading shows pointwise 95% confidence intervals calculated using JKn variance and t(42), and the dotted line denotes aOR=1. The vertical axis is logarithmic, and the curve is displayed for ages 0–100 years. Model specifications, joint tests, representative-age estimates, and age-band sample support are reported in S13 Table.
