# Supplementary material for: Do older vehicles affect occupants of all ages equally? Age-group differences in the association between vehicle age and fatal or suspected serious injury in U.S. police-reported passenger-vehicle crashes, 2016–2024
Source: PLoS One. 2026 Nov 12;21(11):e0362075. doi: 10.1371/journal.pone.0362075 (PMC13759116; doi:10.1371/journal.pone.0362075)
Supplement: Supplementary file 4 [file pone.0362075.s004.docx]

**S1 Table. Variable coding and reference categories for the primary and extended models.**

| **Variable / model** | **Source and analysis variable(s)** | **Analysis coding** | **Reference category** | **Missing-data handling** | **Model use / inference** |
| --- | --- | --- | --- | --- | --- |
| **Primary model variables** | | | | | |
| Vehicle age category | Person.MOD_YEAR; YEAR from annual file vehicle_age_group | 0–4; 5–9; 10–14; 15–19; ≥20 years. vehicle_age = max(0, YEAR − MOD_YEAR). Valid MOD_YEAR: 1900 through YEAR+1. | 0–4 years | Invalid model year excluded before the primary complete-case domain. | M0–M3; all extensions |
| Occupant age group | Person.AGE occupant_age_group | 0–17; 18–44; 45–64; ≥65 years. AGE must be 0–120. | 0–17 years | Unknown/out-of-range age excluded before primary domain. | M1–M3; all extensions |
| Occupant sex | Person.SEX sex_group | 2 → female; 1 → male. | female | Other codes excluded from primary complete cases. | M1–M3; all extensions |
| Seating position | Person.PER_TYP; Person.SEAT_POS position_group | PER_TYP=1 → driver (first priority); otherwise SEAT_POS 12,13,18,19 → front_passenger; 21–49 → rear_passenger; 50–56 → other_position. | driver | Other positions coded unknown at preparation and excluded from primary domain. | M1–M3; all extensions |
| Restraint use | Person.REST_USE; annual-file YEAR restraint_group | 1,2,3,4,6,8,10,11,12,97 → used. None: code 7 in 2016, code 20 in 2017–2024. All other codes → unknown. | used | unknown excluded from primary domain. The factor retains an unused unknown level; no unknown coefficient is estimated in M0–M3. | M2–M3; all extensions |
| Vehicle class | Person.BODY_TYP; Person.TOW_VEH vehicle_class | BODY_TYP 1–11 or 17 → car; 14,15,16,19 → utility; 20,21,22,28,29 → van; 30–39 → pickup; 40,41,45,48,49 or (79 with TOW_VEH 0/9) → other_light. Other codes → ineligible. | car | Passenger-vehicle eligibility applied before main domain. No ineligible class is observed in the primary sample. | M2–M3; all extensions |
| Crash year | Annual CRSS file year, inserted as YEAR year_factor | Categorical factor: 2016,2017,2018,2019,2020,2021,2022,2023,2024. Eight dummy coefficients. | 2016 | Year supplied by annual-file loop; no missing year. | M0–M3; all extensions |
| U.S. census region | Person.REGION region_group | 1 → northeast; 2 → midwest; 3 → south; 4 → west. | northeast | Other/missing codes excluded from primary domain. | M2–M3; all extensions |
| Urbanicity | Person.URBANICITY urbanicity_group | 1 → urban; 2 → rural. | urban | Other/missing codes excluded from primary domain. | M2–M3; all extensions |
| **Driver-characteristics extension** | | | | | |
| Linked driver age | Linked Person.AGE; driver PER_TYP=1 driver_age_group | 0–20 → 0-20; 21–34 → 21-34; 35–64 → 35-64; 65–120 → 65+; other nonmissing values → unknown. | 21-34 | Unmatched driver produces NA, not unknown. NA propagates through ifelse; nonmissing invalid values become unknown. Unknown category in primary sample: n = 1,004. Additional exclusions: 182. | Driver-characteristics extension |
| Linked driver sex | Linked Person.SEX driver_sex_group | 1 → male; 2 → female; other nonmissing values → unknown. | male | Unmatched driver produces NA. Other nonmissing codes remain as the unknown category. Unknown category in primary sample: n = 655. Additional exclusions: 182. | Driver-characteristics extension |
| Driver alcohol involvement | Linked Person.DRINKING and Person.PERALCH_IM driver_drinking_group | If either DRINKING=1 or PERALCH_IM=1 → yes; otherwise if either=0 → no; otherwise unknown. Yes has priority. PERALCH_IM is NHTSA single-imputed. | no | Unmatched driver leaves both source variables NA and final category NA. No unknown category is observed among linked primary-sample records. Unknown category in primary sample: n = 0. Additional exclusions: 182. | Driver-characteristics extension |
| Speeding-related status | Vehicle.SPEEDREL speeding_related_group | 0 → no; 2,3,4,5 → yes; all other nonmissing codes → unknown. | no | unknown retained as a category. No source NA in primary sample. Unknown category in primary sample: n = 9,202. Additional exclusions: 0. | Driver-characteristics extension |
| **Crash-context extension** | | | | | |
| Time of day | Accident.HOUR_IM hour_group | 6–17 inclusive → day; remaining valid hours 0–23 → night; other nonmissing values → unknown. | day | NHTSA single-imputed hour used. Neither NA nor unknown observed in primary sample. Unknown category in primary sample: n = 0. Additional exclusions: 0. | Crash-context extension |
| Lighting conditions | Accident.LGTCON_IM light_group | 1 → daylight; 2 → dark_unlighted; 3 → dark_lighted; 4,5 → dawn_dusk; 6,7 → other; remaining nonmissing codes → unknown. | daylight | NHTSA single-imputed lighting used. Neither NA nor unknown observed in primary sample. Unknown category in primary sample: n = 0. Additional exclusions: 0. | Crash-context extension |
| Weather | Accident.WEATHR_IM weather_group | 1,10 → clear_cloudy; 2,3,4,5,6,7,11,12 → adverse; 8 → other; remaining codes → unknown. | clear_cloudy | NHTSA single-imputed weather used. Neither NA nor unknown observed in primary sample. Unknown category in primary sample: n = 0. Additional exclusions: 0. | Crash-context extension |
| Location relative to trafficway | Accident.REL_ROAD road_group | 1 → roadway; 2 → shoulder; 3–6 → off_roadway; 7,8,10,11,12 → other_trafficway; remaining nonmissing codes → unknown. | roadway | unknown retained as a category. No source NA in primary sample. REL_ROAD does not encode all road characteristics. Unknown category in primary sample: n = 157. Additional exclusions: 0. | Crash-context extension |
| Posted speed limit | Vehicle.VSPD_LIM speed_limit_group | 5–30 → 5_30; 35–45 → 35_45; 50–65 → 50_65; 70–95 → 70_plus; 0 → no_limit_or_nontrafficway; remaining nonmissing codes → unknown. Values are mph codes. | 5_30 | unknown retained as a category. No source NA in primary sample. Unknown category in primary sample: n = 118,631. Additional exclusions: 0. | Crash-context extension |
| **Collision-process extension** | | | | | |
| Manner of collision | Accident.MANCOL_IM collision_group | 0 → not_vehicle_collision; 1 → front_rear; 2 → front_front; 6 → angle; 7,8 → sideswipe; 9,10,11 → other; remaining nonmissing codes → unknown. | not_vehicle_collision | NHTSA single-imputed MANCOL_IM used, not vehicle MAN_COLL. Neither NA nor unknown observed in primary sample. Unknown category in primary sample: n = 0. Additional exclusions: 0. | Collision-process extension |
| Rollover | Vehicle.ROLLOVER rollover_group | 0 → no; 1,2,3 → yes; remaining nonmissing codes → unknown. | no | unknown retained as a category. No source NA in primary sample. Unknown category in primary sample: n = 7,952. Additional exclusions: 0. | Collision-process extension |
| Extent of vehicle damage | Vehicle.DEFORMED damage_group | 0 → none; 2 → minor; 4 → functional; 6 → disabling; remaining nonmissing codes → unknown. | none | unknown retained as a category. No source NA in primary sample. Unknown category in primary sample: n = 172,382. Additional exclusions: 0. | Collision-process extension |
| **Model-specific analytic samples** | | | | | |
| **Model** | **Unweighted N** | **K/A events** | **Excluded records** | **Excluded K/A events** | **Variance; CI and P reference** |
| Primary models M0–M3 | 899,273 | 46,367 | — | — | JKn; t(42) and F(df1,42) |
| driver characteristics | 899,091 | 46,349 | 182 | 18 | Taylor linearization; t(42) |
| crash context | 899,273 | 46,367 | 0 | 0 | Taylor linearization; t(42) |
| collision mechanism | 899,273 | 46,367 | 0 | 0 | Taylor linearization; t(42) |

Note. The 182 records excluded from the driver-characteristics extension are the same unmatched records across the linked driver fields and should not be summed across variables.
