# Supplementary material for: Do older vehicles affect occupants of all ages equally? Age-group differences in the association between vehicle age and fatal or suspected serious injury in U.S. police-reported passenger-vehicle crashes, 2016–2024
Source: PLoS One. 2026 Nov 12;21(11):e0362075. doi: 10.1371/journal.pone.0362075 (PMC13759116; doi:10.1371/journal.pone.0362075)
Supplement: Supplementary file 5 [file pone.0362075.s005.docx]

**S2 Table. Missingness and comparison of included and excluded eligible records.**

**Panel A. Sample-selection stages and missing primary covariates.**

| **Item** | **Unweighted n** | **Survey-weighted proportion (%)** | **Comment** |
| --- | --- | --- | --- |
| All CRSS person records | 1,158,730 | — | Starting sample |
| Eligible passenger-vehicle occupants | 1,015,530 | — | 143,200 records excluded |
| Valid vehicle age, occupant age, and outcome | 951,314 | — | 64,216 records excluded |
| Primary complete-case sample | 899,273 | 94.61 | 52,041 records excluded |
| Missing Sex | 2,887 | 0.31 | Missingness categories may overlap |
| Missing Seating position | 4,551 | 0.46 | Missingness categories may overlap |
| Missing Restraint use | 46,228 | 4.79 | Missingness categories may overlap |
| Missing Census region | 0 | 0.00 | Missingness categories may overlap |
| Missing Urbanicity | 0 | 0.00 | Missingness categories may overlap |
| Missing Any primary covariate | 52,041 | 5.39 | Missingness categories may overlap |

**Panel B. Survey-weighted distributions of included and excluded records.**

| **Variable** | **Category** | **Included in primary analysis, n (%)** | **Excluded, n (%)** |
| --- | --- | --- | --- |
| Crash year | 2016 | 94,378 (12.60) | 4,657 (11.38) |
| Crash year | 2017 | 108,966 (11.76) | 5,110 (9.79) |
| Crash year | 2018 | 95,273 (12.32) | 4,693 (10.54) |
| Crash year | 2019 | 106,942 (12.34) | 5,698 (11.28) |
| Crash year | 2020 | 99,022 (8.80) | 6,381 (9.66) |
| Crash year | 2021 | 103,581 (10.59) | 6,428 (11.41) |
| Crash year | 2022 | 102,046 (10.36) | 6,108 (10.68) |
| Crash year | 2023 | 92,996 (10.64) | 6,325 (12.37) |
| Crash year | 2024 | 96,069 (10.59) | 6,641 (12.88) |
| Vehicle age | 0–4 years | 332,105 (29.87) | 16,681 (25.61) |
| Vehicle age | 5–9 years | 220,673 (27.70) | 12,189 (26.28) |
| Vehicle age | 10–14 years | 179,401 (22.38) | 10,864 (23.11) |
| Vehicle age | 15–19 years | 113,876 (13.78) | 8,106 (16.51) |
| Vehicle age | ≥20 years | 53,218 (6.27) | 4,201 (8.48) |
| Occupant age | 0–17 years | 120,400 (13.32) | 8,068 (15.22) |
| Occupant age | 18–44 years | 478,610 (53.75) | 29,048 (55.90) |
| Occupant age | 45–64 years | 207,213 (22.87) | 10,547 (20.44) |
| Occupant age | ≥65 years | 93,050 (10.06) | 4,378 (8.43) |
| Injury outcome | No fatal or suspected serious injury | 852,906 (98.92) | 47,566 (98.07) |
| Injury outcome | Fatal or suspected serious injury | 46,367 (1.08) | 4,475 (1.93) |

Note. Panel A survey-weighted percentages use the 951,314 records with valid vehicle age, occupant age, and injury outcome as the denominator. In Panel B, counts are unweighted and percentages are survey-weighted.
