# Supplementary material for: Do older vehicles affect occupants of all ages equally? Age-group differences in the association between vehicle age and fatal or suspected serious injury in U.S. police-reported passenger-vehicle crashes, 2016–2024
Source: PLoS One. 2026 Nov 12;21(11):e0362075. doi: 10.1371/journal.pone.0362075 (PMC13759116; doi:10.1371/journal.pone.0362075)
Supplement: Supplementary file 6 [file pone.0362075.s006.docx]

**S3 Table. Characteristics of the primary analysis sample by vehicle age, CRSS 2016–2024.**

| **Characteristic** | **Category** | **Overall** | **0–4 years** | **5–9 years** | **10–14 years** | **15–19 years** | **≥20 years** |
| --- | --- | --- | --- | --- | --- | --- | --- |
| Occupant age, years | 0–17 | 120,400 (13.3) | 41,477 (12.1) | 29,824 (13.4) | 26,486 (14.6) | 16,066 (14.1) | 6,547 (12.2) |
|  | 18–44 | 478,610 (53.8) | 171,327 (52.0) | 119,065 (54.2) | 98,874 (55.4) | 61,925 (54.8) | 27,419 (52.0) |
|  | 45–64 | 207,213 (22.9) | 82,825 (25.2) | 49,103 (22.3) | 37,352 (20.8) | 24,850 (21.6) | 13,083 (24.4) |
|  | ≥65 | 93,050 (10.1) | 36,476 (10.7) | 22,681 (10.0) | 16,689 (9.2) | 11,035 (9.5) | 6,169 (11.4) |
| Sex | Female | 432,871 (47.8) | 173,201 (51.6) | 112,601 (51.5) | 84,666 (47.4) | 45,872 (40.5) | 16,531 (30.9) |
|  | Male | 466,402 (52.2) | 158,904 (48.4) | 108,072 (48.5) | 94,735 (52.6) | 68,004 (59.5) | 36,687 (69.1) |
| Seating position | Driver | 662,454 (75.3) | 238,996 (73.9) | 163,713 (75.2) | 132,760 (75.3) | 85,624 (76.5) | 41,361 (79.5) |
|  | Front passenger | 134,794 (14.2) | 51,719 (14.5) | 31,596 (13.8) | 26,533 (14.1) | 16,980 (14.2) | 7,966 (13.9) |
|  | Rear passenger | 101,260 (10.5) | 41,123 (11.5) | 25,200 (11.0) | 19,959 (10.5) | 11,166 (9.3) | 3,812 (6.5) |
|  | Other position | 765 (0.1) | 267 (0.1) | 164 (0.1) | 149 (0.1) | 106 (0.1) | 79 (0.1) |
| Restraint use | Used | 870,890 (98.0) | 325,114 (98.7) | 215,125 (98.4) | 172,948 (97.8) | 108,144 (97.1) | 49,559 (96.1) |
|  | None | 28,383 (2.0) | 6,991 (1.3) | 5,548 (1.6) | 6,453 (2.2) | 5,732 (2.9) | 3,659 (3.9) |
| Vehicle class | Passenger car | 471,427 (53.1) | 159,573 (47.8) | 128,791 (58.5) | 103,335 (57.7) | 56,522 (50.4) | 23,206 (44.3) |
|  | Utility vehicle | 235,426 (25.4) | 107,201 (32.1) | 51,807 (23.6) | 39,262 (21.9) | 27,047 (23.2) | 10,109 (18.7) |
|  | Van | 48,581 (5.5) | 16,338 (5.1) | 11,206 (5.1) | 11,036 (6.1) | 7,280 (6.4) | 2,721 (5.0) |
|  | Pickup | 113,784 (12.9) | 33,796 (10.6) | 21,888 (9.9) | 21,539 (12.1) | 20,368 (17.8) | 16,193 (30.2) |
|  | Other light vehicle | 30,055 (3.1) | 15,197 (4.4) | 6,981 (3.0) | 4,229 (2.3) | 2,659 (2.3) | 989 (1.8) |
| Urbanicity | Urban | 701,547 (77.4) | 269,109 (80.9) | 173,203 (78.0) | 137,554 (76.2) | 84,718 (74.2) | 36,963 (69.5) |
|  | Rural | 197,726 (22.6) | 62,996 (19.1) | 47,470 (22.0) | 41,847 (23.8) | 29,158 (25.8) | 16,255 (30.5) |
| Injury outcome | No fatal or suspected serious injury | 852,906 (98.9) | 318,568 (99.3) | 211,362 (99.1) | 169,477 (98.8) | 105,405 (98.4) | 48,094 (97.9) |
|  | Fatal or suspected serious injury | 46,367 (1.1) | 13,537 (0.7) | 9,311 (0.9) | 9,924 (1.2) | 8,471 (1.6) | 5,124 (2.1) |

Note. Values are unweighted counts with survey-weighted column percentages.
