# Supplementary material for: Do older vehicles affect occupants of all ages equally? Age-group differences in the association between vehicle age and fatal or suspected serious injury in U.S. police-reported passenger-vehicle crashes, 2016–2024
Source: PLoS One. 2026 Nov 12;21(11):e0362075. doi: 10.1371/journal.pone.0362075 (PMC13759116; doi:10.1371/journal.pone.0362075)
Supplement: Supplementary file 7 [file pone.0362075.s007.docx]

**S4 Table. Seating-position composition within occupant age groups.**

| **Occupant age (years)** | **Seating position** | **Unweighted n** | **Survey-weighted row percentage (%)** |
| --- | --- | --- | --- |
| 0–17 | Driver | 23,259 | 21.9 |
| 0–17 | Front passenger | 25,517 | 21.0 |
| 0–17 | Rear passenger | 71,254 | 56.9 |
| 0–17 | Other position | 370 | 0.3 |
| 18–44 | Driver | 387,614 | 82.5 |
| 18–44 | Front passenger | 69,341 | 13.5 |
| 18–44 | Rear passenger | 21,404 | 3.9 |
| 18–44 | Other position | 251 | 0.0 |
| 45–64 | Driver | 176,357 | 86.2 |
| 45–64 | Front passenger | 25,073 | 11.3 |
| 45–64 | Rear passenger | 5,692 | 2.4 |
| 45–64 | Other position | 91 | 0.0 |
| ≥65 | Driver | 75,224 | 82.2 |
| ≥65 | Front passenger | 14,863 | 14.9 |
| ≥65 | Rear passenger | 2,910 | 2.8 |
| ≥65 | Other position | 53 | 0.1 |
