# Supplementary material for: Do older vehicles affect occupants of all ages equally? Age-group differences in the association between vehicle age and fatal or suspected serious injury in U.S. police-reported passenger-vehicle crashes, 2016–2024
Source: PLoS One. 2026 Nov 12;21(11):e0362075. doi: 10.1371/journal.pone.0362075 (PMC13759116; doi:10.1371/journal.pone.0362075)
Supplement: Supplementary file 8 [file pone.0362075.s008.docx]

**S5 Table. Sample support and injury proportions within occupant-age-by-vehicle-age cells.**

| **Occupant age (years)** | **Vehicle age (years)** | **Unweighted n** | **Unweighted K/A events** | **Weighted K/A (%)** | **95% CI (%)** |
| --- | --- | --- | --- | --- | --- |
| 0–17 | 0–4 | 41,477 | 867 | 0.40 | 0.34–0.45 |
| 0–17 | 5–9 | 29,824 | 737 | 0.49 | 0.41–0.57 |
| 0–17 | 10–14 | 26,486 | 922 | 0.76 | 0.65–0.87 |
| 0–17 | 15–19 | 16,066 | 778 | 1.11 | 0.93–1.28 |
| 0–17 | ≥20 | 6,547 | 442 | 1.58 | 1.34–1.82 |
| 18–44 | 0–4 | 171,327 | 7,046 | 0.69 | 0.62–0.77 |
| 18–44 | 5–9 | 119,065 | 5,051 | 0.84 | 0.74–0.94 |
| 18–44 | 10–14 | 98,874 | 5,669 | 1.21 | 1.07–1.35 |
| 18–44 | 15–19 | 61,925 | 4,817 | 1.66 | 1.46–1.85 |
| 18–44 | ≥20 | 27,419 | 2,730 | 2.17 | 1.97–2.37 |
| 45–64 | 0–4 | 82,825 | 3,519 | 0.76 | 0.65–0.86 |
| 45–64 | 5–9 | 49,103 | 2,132 | 0.89 | 0.77–1.00 |
| 45–64 | 10–14 | 37,352 | 2,186 | 1.25 | 1.10–1.40 |
| 45–64 | 15–19 | 24,850 | 1,963 | 1.75 | 1.53–1.97 |
| 45–64 | ≥20 | 13,083 | 1,306 | 2.20 | 1.91–2.49 |
| ≥65 | 0–4 | 36,476 | 2,105 | 1.09 | 0.97–1.21 |
| ≥65 | 5–9 | 22,681 | 1,391 | 1.35 | 1.18–1.51 |
| ≥65 | 10–14 | 16,689 | 1,147 | 1.62 | 1.38–1.87 |
| ≥65 | 15–19 | 11,035 | 913 | 1.92 | 1.59–2.25 |
| ≥65 | ≥20 | 6,169 | 646 | 2.56 | 2.23–2.89 |
