# Supplementary material for: Do older vehicles affect occupants of all ages equally? Age-group differences in the association between vehicle age and fatal or suspected serious injury in U.S. police-reported passenger-vehicle crashes, 2016–2024
Source: PLoS One. 2026 Nov 12;21(11):e0362075. doi: 10.1371/journal.pone.0362075 (PMC13759116; doi:10.1371/journal.pone.0362075)
Supplement: Supplementary file 9 [file pone.0362075.s009.docx]

**S6 Table. Survey-weighted proportion of fatal or suspected serious injury by vehicle-age category.**

| **Vehicle age (years)** | **Unweighted n** | **Unweighted K/A events** | **Survey-weighted K/A proportion (%)** | **95% CI (%)** |
| --- | --- | --- | --- | --- |
| 0–4 | 332,105 | 13,537 | 0.72 | 0.64–0.80 |
| 5–9 | 220,673 | 9,311 | 0.86 | 0.75–0.96 |
| 10–14 | 179,401 | 9,924 | 1.19 | 1.05–1.33 |
| 15–19 | 113,876 | 8,471 | 1.62 | 1.43–1.82 |
| ≥20 | 53,218 | 5,124 | 2.15 | 1.93–2.36 |
