# Supplementary material for: Do older vehicles affect occupants of all ages equally? Age-group differences in the association between vehicle age and fatal or suspected serious injury in U.S. police-reported passenger-vehicle crashes, 2016–2024
Source: PLoS One. 2026 Nov 12;21(11):e0362075. doi: 10.1371/journal.pone.0362075 (PMC13759116; doi:10.1371/journal.pone.0362075)
Supplement: Supplementary file 10 [file pone.0362075.s010.docx]

**S7 Table. Pairwise comparisons of age-specific adjusted odds ratios for vehicles aged ≥20 versus 0–4 years.**

| **Age-group comparison** | **Ratio of ORs** | **95% CI** | **Raw P** | **Holm-adjusted P** |
| --- | --- | --- | --- | --- |
| 0–17 vs 18–44 | 1.170 | 0.949–1.442 | 0.1383 | 0.4064 |
| 0–17 vs 45–64 | 1.212 | 0.939–1.565 | 0.1355 | 0.4064 |
| 0–17 vs ≥65 | 1.407 | 1.109–1.784 | 0.0059 | 0.0354 |
| 18–44 vs 45–64 | 1.036 | 0.934–1.151 | 0.4930 | 0.4930 |
| 18–44 vs ≥65 | 1.203 | 1.049–1.379 | 0.0094 | 0.0469 |
| 45–64 vs ≥65 | 1.160 | 0.998–1.350 | 0.0535 | 0.2142 |

Note. Ratios >1 indicate a larger relative association in the first-listed age group.
