# Supplementary material for: Do older vehicles affect occupants of all ages equally? Age-group differences in the association between vehicle age and fatal or suspected serious injury in U.S. police-reported passenger-vehicle crashes, 2016–2024
Source: PLoS One. 2026 Nov 12;21(11):e0362075. doi: 10.1371/journal.pone.0362075 (PMC13759116; doi:10.1371/journal.pone.0362075)
Supplement: Supplementary file 11 [file pone.0362075.s011.docx]

**S8 Table. Comparison of JKn replicate-weight and Taylor linearization estimates.**

**Panel A. Comparison of core joint tests.**

| **Test** | **Variance method** | **F** | **df1** | **df2** | **P** |
| --- | --- | --- | --- | --- | --- |
| Complete vehicle-age-by-occupant-age interaction | JKn | 2.167 | 12 | 42 | 0.0323 |
| Complete vehicle-age-by-occupant-age interaction | Taylor | 2.174 | 12 | 42 | 0.0318 |
| Equality of ≥20-year versus 0–4-year log aORs across age groups | JKn | 3.642 | 3 | 42 | 0.0201 |
| Equality of ≥20-year versus 0–4-year log aORs across age groups | Taylor | 3.674 | 3 | 42 | 0.0194 |

**Panel B. Comparison of core effect estimates.**

| **Result** | **JKn aOR (95% CI)** | **Taylor aOR (95% CI)** | **SE ratio (JKn/Taylor)** |
| --- | --- | --- | --- |
| Overall: 5–9 years vs 0–4 years | 1.14 (1.09–1.19) | 1.14 (1.09–1.19) | 1.004 |
| Overall: 10–14 years vs 0–4 years | 1.50 (1.43–1.57) | 1.50 (1.43–1.57) | 1.009 |
| Overall: 15–19 years vs 0–4 years | 1.94 (1.84–2.05) | 1.94 (1.84–2.05) | 1.011 |
| Overall: ≥20 years vs 0–4 years | 2.34 (2.19–2.49) | 2.34 (2.19–2.49) | 1.011 |
| 0–17 years: ≥20 years vs 0–4 years | 2.80 (2.27–3.44) | 2.80 (2.28–3.44) | 1.005 |
| 18–44 years: ≥20 years vs 0–4 years | 2.39 (2.23–2.57) | 2.39 (2.23–2.57) | 1.012 |
| 45–64 years: ≥20 years vs 0–4 years | 2.31 (2.07–2.57) | 2.31 (2.07–2.57) | 1.002 |
| ≥65 years: ≥20 years vs 0–4 years | 1.99 (1.75–2.26) | 1.99 (1.75–2.26) | 1.009 |

Note. The SE ratio is the JKn standard error divided by the Taylor standard error for the corresponding log(aOR) contrast.
