# Supplementary material for: Do older vehicles affect occupants of all ages equally? Age-group differences in the association between vehicle age and fatal or suspected serious injury in U.S. police-reported passenger-vehicle crashes, 2016–2024
Source: PLoS One. 2026 Nov 12;21(11):e0362075. doi: 10.1371/journal.pone.0362075 (PMC13759116; doi:10.1371/journal.pone.0362075)
Supplement: Supplementary file 12 [file pone.0362075.s012.docx]

**S9 Table. Standardized predicted probabilities, probability differences, and age-group comparisons.**

**Panel A. Predicted probabilities standardized to a common covariate distribution.**

| **Occupant age (years)** | **Vehicle age (years)** | **Standardized predicted probability (%)** | **95% CI (%)** |
| --- | --- | --- | --- |
| 0–17 | 0–4 | 0.45 | 0.39–0.51 |
| 0–17 | 5–9 | 0.52 | 0.44–0.59 |
| 0–17 | 10–14 | 0.73 | 0.62–0.84 |
| 0–17 | 15–19 | 0.97 | 0.82–1.11 |
| 0–17 | ≥20 | 1.22 | 0.99–1.45 |
| 18–44 | 0–4 | 0.74 | 0.68–0.81 |
| 18–44 | 5–9 | 0.85 | 0.76–0.94 |
| 18–44 | 10–14 | 1.11 | 1.00–1.22 |
| 18–44 | 15–19 | 1.41 | 1.27–1.56 |
| 18–44 | ≥20 | 1.71 | 1.55–1.87 |
| 45–64 | 0–4 | 0.89 | 0.79–1.00 |
| 45–64 | 5–9 | 0.99 | 0.88–1.10 |
| 45–64 | 10–14 | 1.32 | 1.18–1.46 |
| 45–64 | 15–19 | 1.75 | 1.56–1.94 |
| 45–64 | ≥20 | 1.98 | 1.73–2.23 |
| ≥65 | 0–4 | 1.23 | 1.09–1.38 |
| ≥65 | 5–9 | 1.44 | 1.27–1.61 |
| ≥65 | 10–14 | 1.67 | 1.41–1.93 |
| ≥65 | 15–19 | 1.98 | 1.63–2.32 |
| ≥65 | ≥20 | 2.36 | 2.01–2.70 |

**Panel B. Standardized probability differences for vehicles aged ≥20 years minus 0–4 years.**

| **Occupant age (years)** | **Probability difference (percentage points)** | **95% CI (percentage points)** | **P** |
| --- | --- | --- | --- |
| 0–17 | 0.77 | 0.55–0.99 | <0.001 |
| 18–44 | 0.97 | 0.85–1.09 | <0.001 |
| 45–64 | 1.08 | 0.89–1.28 | <0.001 |
| ≥65 | 1.12 | 0.84–1.40 | <0.001 |

**Panel C. Age-group equality test and pairwise comparisons of standardized probability differences.**

| **Omnibus test** | | **Wald χ²** | | **F** | **df1** | | **df2** | | **P** |
| --- | --- | --- | --- | --- | --- | --- | --- | --- | --- |
| Equality of probability differences across four age groups | | 7.237 | | 2.412 | 3 | | 42 | | 0.080 |
| **Age-group comparison** | **Difference in probability differences (percentage points)** | | **95% CI (percentage points)** | | | **Raw P** | | **Holm-adjusted P** | |
| 0–17 minus 18–44 | -0.196 | | -0.397–0.005 | | | 0.055 | | 0.221 | |
| 0–17 minus 45–64 | -0.313 | | -0.587–-0.038 | | | 0.027 | | 0.138 | |
| 0–17 minus ≥65 | -0.351 | | -0.650–-0.051 | | | 0.023 | | 0.138 | |
| 18–44 minus 45–64 | -0.117 | | -0.291–0.058 | | | 0.185 | | 0.555 | |
| 18–44 minus ≥65 | -0.154 | | -0.419–0.110 | | | 0.246 | | 0.555 | |
| 45–64 minus ≥65 | -0.038 | | -0.325–0.250 | | | 0.792 | | 0.792 | |
