# Supplementary material for: Do older vehicles affect occupants of all ages equally? Age-group differences in the association between vehicle age and fatal or suspected serious injury in U.S. police-reported passenger-vehicle crashes, 2016–2024
Source: PLoS One. 2026 Nov 12;21(11):e0362075. doi: 10.1371/journal.pone.0362075 (PMC13759116; doi:10.1371/journal.pone.0362075)
Supplement: Supplementary file 13 [file pone.0362075.s013.docx]

**S10 Table. Sensitivity analyses of the overall association comparing vehicles aged ≥20 with those aged 0–4 years.**

**Panel A. Robustness to alternative analysis settings.**

| **Analysis** | **Sample size** | **K/A events** | **aOR (95% CI)** | **P** | **Sample restriction or adjustment-set note** |
| --- | --- | --- | --- | --- | --- |
| Primary overall-association model (Model 2) | 899,273 | 46,367 | 2.34 (2.19–2.49) | <0.001 | Primary complete cases; main adjustment set |
| Crashes in 2019–2024 only | 600,656 | 31,019 | 2.29 (2.13–2.46) | <0.001 | Restricted to 2019–2024 |
| NHTSA-imputed variables | 923,862 | 47,292 | 2.31 (2.17–2.46) | <0.001 | NHTSA single-imputation variables |
| Missing values retained as separate categories | 951,314 | 50,842 | 2.32 (2.19–2.47) | <0.001 | Descriptive survey domain; missing categories retained |
| Vehicle age ≤40 years | 898,621 | 46,260 | 2.33 (2.19–2.48) | <0.001 | Vehicle age >40 years excluded |
| Suspected serious injury only (A) | 893,003 | 40,097 | 2.29 (2.14–2.45) | <0.001 | Fatal injuries excluded; outcome restricted to A-level injury |
| Restrained occupants only | 870,890 | 35,361 | 2.33 (2.17–2.50) | <0.001 | Restricted to restrained occupants |

**Panel B. Alternative covariate adjustment sets.**

| **Analysis** | **Sample size** | **K/A events** | **aOR (95% CI)** | **P** | **Sample restriction or adjustment-set note** |
| --- | --- | --- | --- | --- | --- |
| Without adjustment for restraint use | 899,273 | 46,367 | 2.88 (2.69–3.09) | <0.001 | Primary sample; restraint use omitted from adjustment |
| Driver-characteristics extended model | 899,091 | 46,349 | 2.17 (2.03–2.32) | <0.001 | Separate driver-characteristics extension; 182 unmatched driver links excluded (18 K/A events) |
| Crash-context extended model | 899,273 | 46,367 | 2.09 (1.99–2.20) | <0.001 | Separate crash-context extension; categorical unknown values retained where defined |
| Collision-mechanism extended model | 899,273 | 46,367 | 1.60 (1.50–1.69) | <0.001 | Separate extension for manner of collision, rollover, and extent of vehicle damage |

**Panel C. Vehicle-class-specific analyses.**

| **Analysis** | **Sample size** | **K/A events** | **aOR (95% CI)** | **P** | **Sample restriction or adjustment-set note** |
| --- | --- | --- | --- | --- | --- |
| Passenger cars | 471,427 | 26,456 | 2.11 (1.97–2.26) | <0.001 | Restricted to passenger cars |
| Utility vehicles | 235,426 | 10,617 | 2.86 (2.52–3.24) | <0.001 | Restricted to utility vehicles |
| Vans | 48,581 | 1,899 | 2.46 (1.88–3.22) | <0.001 | Restricted to vans |
| Pickup trucks | 113,784 | 6,176 | 2.44 (2.17–2.75) | <0.001 | Restricted to pickup trucks |

Note. Sample sizes and K/A event counts are unweighted. The primary estimate uses JKn variance and all other estimates use Taylor linearization. Confidence intervals use t(42) for the primary, missing-category, and extended-adjustment analyses and a normal reference for the remaining analyses; reported P values use t(42).
