# Supplementary material for: Do older vehicles affect occupants of all ages equally? Age-group differences in the association between vehicle age and fatal or suspected serious injury in U.S. police-reported passenger-vehicle crashes, 2016–2024
Source: PLoS One. 2026 Nov 12;21(11):e0362075. doi: 10.1371/journal.pone.0362075 (PMC13759116; doi:10.1371/journal.pone.0362075)
Supplement: Supplementary file 14 [file pone.0362075.s014.docx]

**S11 Table. Continuous vehicle-age spline estimates relative to vehicle age 0 years.**

| **Vehicle age (years)** | **aOR** | **95% CI** | **P** |
| --- | --- | --- | --- |
| 0 | 1.00 (reference) | — | — |
| 5 | 1.11 | 1.06–1.17 | <0.001 |
| 10 | 1.18 | 1.11–1.26 | <0.001 |
| 20 | 1.67 | 1.57–1.77 | <0.001 |
| 30 | 2.69 | 2.50–2.89 | <0.001 |
| 40 | 2.73 | 2.12–3.50 | <0.001 |

Note. Other adjustment variables were as in Model 2.
