# Supplementary material for: Do older vehicles affect occupants of all ages equally? Age-group differences in the association between vehicle age and fatal or suspected serious injury in U.S. police-reported passenger-vehicle crashes, 2016–2024
Source: PLoS One. 2026 Nov 12;21(11):e0362075. doi: 10.1371/journal.pone.0362075 (PMC13759116; doi:10.1371/journal.pone.0362075)
Supplement: Supplementary file 15 [file pone.0362075.s015.docx]

**S12 Table. Robustness analyses of age-group heterogeneity.**

| **Analysis** | **Sample size** | **K/A events** | **F** | **df1** | **df2** | **P** | **Test or restriction** |
| --- | --- | --- | --- | --- | --- | --- | --- |
| Primary analysis (Taylor linearization) | 899,273 | 46,367 | 2.174 | 12 | 42 | 0.032 | Complete vehicle-age-by-age-group interaction |
| 2019–2024 only | 600,656 | 31,019 | 3.774 | 12 | 42 | <0.001 | Complete vehicle-age-by-age-group interaction |
| NHTSA-imputed variables | 923,862 | 47,292 | 2.077 | 12 | 42 | 0.040 | Complete vehicle-age-by-age-group interaction |
| Restrained occupants only | 870,890 | 35,361 | 3.000 | 12 | 42 | 0.004 | Complete vehicle-age-by-age-group interaction |
| Drivers only | 662,454 | 35,063 | 1.803 | 12 | 42 | 0.079 | Complete vehicle-age-by-age-group interaction |
| Adults only | 778,873 | 42,621 | 1.844 | 8 | 42 | 0.095 | Complete vehicle-age-by-age-group interaction |
| Vehicle age ≤40 years | 898,621 | 46,260 | 2.205 | 12 | 42 | 0.029 | Complete vehicle-age-by-age-group interaction |
| Minors vs adults (Taylor) | 899,273 | 46,367 | 1.832 | 4 | 42 | 0.141 | Vehicle-age-by-binary-age interaction |
| Minors vs adults (JKn) | 899,273 | 46,367 | 1.834 | 4 | 42 | 0.140 | Vehicle-age-by-binary-age interaction |
| Age subgroups within 0–17 years | 120,400 | 3,746 | 1.788 | 8 | 42 | 0.107 | Age groups 0–8, 9–15, and 16–17 years |
