# Supplementary material for: Do older vehicles affect occupants of all ages equally? Age-group differences in the association between vehicle age and fatal or suspected serious injury in U.S. police-reported passenger-vehicle crashes, 2016–2024
Source: PLoS One. 2026 Nov 12;21(11):e0362075. doi: 10.1371/journal.pone.0362075 (PMC13759116; doi:10.1371/journal.pone.0362075)
Supplement: Supplementary file 16 [file pone.0362075.s016.docx]

**S13 Table. Continuous occupant-age spline models, representative estimates, and sample support.**

**Panel A. Model specifications and joint interaction tests.**

| **Occupant-age spline df** | **Internal knots, years** | **Boundary knots, years** | **F** | **df1** | **df2** | **P** |
| --- | --- | --- | --- | --- | --- | --- |
| 3 | 25, 45 | 0, 120 | 3.412 | 12 | 42 | 0.0015 |
| 4 | 22, 34, 51 | 0, 120 | 2.842 | 16 | 42 | 0.0034 |

**Panel B. Representative-age estimates.**

| **Occupant age (years)** | **df3 aOR (95% CI)** | **df4 aOR (95% CI)** |
| --- | --- | --- |
| 5 | 2.68 (2.03–3.53) | 3.08 (2.10–4.52) |
| 10 | 2.58 (2.14–3.12) | 2.67 (2.14–3.33) |
| 15 | 2.50 (2.21–2.83) | 2.40 (2.14–2.69) |
| 18 | 2.47 (2.24–2.72) | 2.31 (2.10–2.54) |
| 25 | 2.42 (2.22–2.63) | 2.33 (2.13–2.54) |
| 35 | 2.44 (2.24–2.65) | 2.63 (2.36–2.93) |
| 45 | 2.46 (2.22–2.71) | 2.55 (2.29–2.84) |
| 55 | 2.37 (2.12–2.64) | 2.29 (2.06–2.55) |
| 65 | 2.18 (1.98–2.40) | 2.08 (1.88–2.30) |
| 75 | 1.93 (1.73–2.15) | 1.91 (1.71–2.14) |
| 85 | 1.65 (1.37–2.00) | 1.78 (1.46–2.17) |

**Panel C. Sample support by five-year age band.**

| **Occupant age** | **N** | **K/A events** | **0–4-year vehicles N** | **0–4-year vehicles events** | **≥20-year vehicles N** | **≥20-year vehicles events** |
| --- | --- | --- | --- | --- | --- | --- |
| 0–4 | 26,654 | 465 | 10,251 | 135 | 960 | 39 |
| 5–9 | 25,018 | 621 | 9,806 | 158 | 916 | 52 |
| 10–14 | 24,836 | 772 | 9,783 | 210 | 1,075 | 77 |
| 15–19 | 93,402 | 4,345 | 24,239 | 802 | 7,561 | 638 |
| 20–24 | 113,226 | 6,090 | 36,983 | 1,686 | 6,922 | 646 |
| 25–29 | 96,833 | 5,266 | 36,293 | 1,477 | 5,064 | 508 |
| 30–34 | 82,843 | 4,506 | 32,032 | 1,327 | 4,230 | 475 |
| 35–39 | 72,401 | 3,701 | 28,036 | 1,100 | 3,707 | 354 |
| 40–44 | 63,797 | 3,293 | 25,381 | 1,018 | 3,531 | 383 |
| 45–49 | 58,213 | 3,023 | 23,807 | 931 | 3,262 | 324 |
| 50–54 | 54,556 | 2,902 | 21,884 | 924 | 3,329 | 343 |
| 55–59 | 50,761 | 2,788 | 20,036 | 914 | 3,388 | 331 |
| 60–64 | 43,683 | 2,393 | 17,098 | 750 | 3,104 | 308 |
| 65–69 | 33,709 | 1,941 | 13,493 | 651 | 2,262 | 230 |
| 70–74 | 25,420 | 1,640 | 10,290 | 582 | 1,632 | 163 |
| 75–79 | 17,052 | 1,175 | 6,693 | 419 | 1,061 | 101 |
| 80–84 | 9,993 | 808 | 3,700 | 263 | 695 | 89 |
| 85–89 | 4,949 | 443 | 1,675 | 126 | 358 | 38 |
| 90–94 | 1,531 | 167 | 511 | 54 | 132 | 23 |
| 95–99 | 320 | 25 | 92 | 9 | 24 | 2 |
| ≥100 | 76 | 3 | 22 | 1 | 5 | 0 |

Note. Representative ages in Panel B are descriptive points on the fitted curves rather than separate hypothesis tests. Panel C reports unweighted sample support. S2 Fig shows the continuous-age curve from the 3-df model.
