# Supplementary material for: Do older vehicles affect occupants of all ages equally? Age-group differences in the association between vehicle age and fatal or suspected serious injury in U.S. police-reported passenger-vehicle crashes, 2016–2024
Source: PLoS One. 2026 Nov 12;21(11):e0362075. doi: 10.1371/journal.pone.0362075 (PMC13759116; doi:10.1371/journal.pone.0362075)
Supplement: Supplementary file 17 [file pone.0362075.s017.docx]

**S14 Table. Vehicle-age associations and age heterogeneity by occupant role.**

**Panel A. Vehicle-age associations by driver/passenger role.**

| **Result type** | **Group or contrast** | **aOR or ratio (95% CI)** | **F** | **df1** | **df2** | **P** | **Holm P** |
| --- | --- | --- | --- | --- | --- | --- | --- |
| Role-specific association | Driver: 5–9 vs 0–4 years | 1.13 (1.08–1.18) | — | — | — | — | — |
| Role-specific association | Driver: 10–14 vs 0–4 years | 1.49 (1.41–1.57) | — | — | — | — | — |
| Role-specific association | Driver: 15–19 vs 0–4 years | 1.94 (1.83–2.07) | — | — | — | — | — |
| Role-specific association | Driver: 20+ vs 0–4 years | 2.27 (2.11–2.44) | — | — | — | — | — |
| Role-specific association | Passenger: 5–9 vs 0–4 years | 1.18 (1.07–1.30) | — | — | — | — | — |
| Role-specific association | Passenger: 10–14 vs 0–4 years | 1.55 (1.43–1.69) | — | — | — | — | — |
| Role-specific association | Passenger: 15–19 vs 0–4 years | 1.97 (1.81–2.15) | — | — | — | — | — |
| Role-specific association | Passenger: 20+ vs 0–4 years | 2.66 (2.40–2.96) | — | — | — | — | — |
| Joint test | Vehicle age category × role | — | 2.29 | 4 | 42 | 0.076 | — |
| Joint test | Equality of 20+ vs 0–4 log(aOR) across roles | — | 7.68 | 1 | 42 | 0.008 | — |
| Pairwise contrast | Driver vs passenger, 20+ vs 0–4 | 0.85 (0.76–0.96) | — | — | — | 0.008 | 0.008 |

**Panel B. Age heterogeneity within driver and passenger strata.**

**Drivers**

| **Result type** | **Group or contrast** | **aOR or ratio (95% CI)** | **F** | **df1** | **df2** | **P** | **Holm P** |
| --- | --- | --- | --- | --- | --- | --- | --- |
| Age-specific association | 0–17 years: 20+ vs 0–4 | 1.85 (1.32–2.58) | — | — | — | — | — |
| Age-specific association | 18–44 years: 20+ vs 0–4 | 2.36 (2.17–2.57) | — | — | — | — | — |
| Age-specific association | 45–64 years: 20+ vs 0–4 | 2.30 (2.08–2.54) | — | — | — | — | — |
| Age-specific association | 65+ years: 20+ vs 0–4 | 2.03 (1.77–2.33) | — | — | — | — | — |
| Joint test | Complete vehicle age category × age group interaction | — | 1.80 | 12 | 42 | 0.081 | — |
| Joint test | Equality of 20+ vs 0–4 log(aOR) across age groups | — | 1.63 | 3 | 42 | 0.196 | — |
| Pairwise contrast | 0–17 vs 18–44 | 0.78 (0.56–1.09) | — | — | — | 0.146 | 0.628 |
| Pairwise contrast | 0–17 vs 45–64 | 0.80 (0.57–1.14) | — | — | — | 0.212 | 0.636 |
| Pairwise contrast | 0–17 vs 65+ | 0.91 (0.66–1.26) | — | — | — | 0.554 | 1.000 |
| Pairwise contrast | 18–44 vs 45–64 | 1.03 (0.93–1.14) | — | — | — | 0.569 | 1.000 |
| Pairwise contrast | 18–44 vs 65+ | 1.16 (1.00–1.35) | — | — | — | 0.051 | 0.306 |
| Pairwise contrast | 45–64 vs 65+ | 1.13 (0.97–1.32) | — | — | — | 0.126 | 0.628 |

**Passengers**

| **Result type** | **Group or contrast** | **aOR or ratio (95% CI)** | **F** | **df1** | **df2** | **P** | **Holm P** |
| --- | --- | --- | --- | --- | --- | --- | --- |
| Age-specific association | 0–17 years: 20+ vs 0–4 | 2.98 (2.35–3.79) | — | — | — | — | — |
| Age-specific association | 18–44 years: 20+ vs 0–4 | 2.51 (2.16–2.91) | — | — | — | — | — |
| Age-specific association | 45–64 years: 20+ vs 0–4 | 2.37 (1.83–3.06) | — | — | — | — | — |
| Age-specific association | 65+ years: 20+ vs 0–4 | 2.05 (1.55–2.71) | — | — | — | — | — |
| Joint test | Complete vehicle age category × age group interaction | — | 1.84 | 12 | 42 | 0.072 | — |
| Joint test | Equality of 20+ vs 0–4 log(aOR) across age groups | — | 1.30 | 3 | 42 | 0.287 | — |
| Pairwise contrast | 0–17 vs 18–44 | 1.19 (0.87–1.63) | — | — | — | 0.274 | 1.000 |
| Pairwise contrast | 0–17 vs 45–64 | 1.26 (0.83–1.91) | — | — | — | 0.263 | 1.000 |
| Pairwise contrast | 0–17 vs 65+ | 1.46 (0.98–2.16) | — | — | — | 0.060 | 0.363 |
| Pairwise contrast | 18–44 vs 45–64 | 1.06 (0.81–1.38) | — | — | — | 0.657 | 1.000 |
| Pairwise contrast | 18–44 vs 65+ | 1.22 (0.93–1.61) | — | — | — | 0.142 | 0.712 |
| Pairwise contrast | 45–64 vs 65+ | 1.15 (0.84–1.59) | — | — | — | 0.373 | 1.000 |

Note. Pairwise contrasts are ratios of the first-listed to the second-listed aORs for the ≥20-versus-0–4-year comparison. Holm adjustment was applied separately within the driver and passenger strata.
