# Supplementary material for: Do older vehicles affect occupants of all ages equally? Age-group differences in the association between vehicle age and fatal or suspected serious injury in U.S. police-reported passenger-vehicle crashes, 2016–2024
Source: PLoS One. 2026 Nov 12;21(11):e0362075. doi: 10.1371/journal.pone.0362075 (PMC13759116; doi:10.1371/journal.pone.0362075)
Supplement: Supplementary file 18 [file pone.0362075.s018.docx]

**S15 Table. Age-group heterogeneity after within-group continuous-age adjustment.**

| **Result type** | **Group or contrast** | **aOR or ratio (95% CI)** | **F** | **df1** | **df2** | **P** | **Holm P** |
| --- | --- | --- | --- | --- | --- | --- | --- |
| Age-specific association | 0–17 years: 20+ vs 0–4 | 2.58 (2.09–3.19) | — | — | — | — | — |
| Age-specific association | 18–44 years: 20+ vs 0–4 | 2.43 (2.26–2.61) | — | — | — | — | — |
| Age-specific association | 45–64 years: 20+ vs 0–4 | 2.31 (2.07–2.57) | — | — | — | — | — |
| Age-specific association | 65+ years: 20+ vs 0–4 | 1.97 (1.74–2.24) | — | — | — | — | — |
| Joint test | Complete vehicle age category × age group interaction | — | 1.78 | 12 | 42 | 0.083 | — |
| Joint test | Equality of 20+ vs 0–4 log(aOR) across age groups | — | 3.48 | 3 | 42 | 0.024 | — |
| Pairwise contrast | 0–17 vs 18–44 | 1.06 (0.86–1.32) | — | — | — | 0.559 | 1.000 |
| Pairwise contrast | 0–17 vs 45–64 | 1.12 (0.86–1.45) | — | — | — | 0.387 | 1.000 |
| Pairwise contrast | 0–17 vs 65+ | 1.31 (1.03–1.67) | — | — | — | 0.029 | 0.144 |
| Pairwise contrast | 18–44 vs 45–64 | 1.05 (0.95–1.17) | — | — | — | 0.337 | 1.000 |
| Pairwise contrast | 18–44 vs 65+ | 1.23 (1.07–1.41) | — | — | — | 0.004 | 0.021 |
| Pairwise contrast | 45–64 vs 65+ | 1.17 (1.01–1.36) | — | — | — | 0.040 | 0.159 |

Note. Pairwise contrasts are ratios of the first-listed to the second-listed age-specific aORs for the ≥20-versus-0–4-year comparison.
