# Supplementary material for: Do older vehicles affect occupants of all ages equally? Age-group differences in the association between vehicle age and fatal or suspected serious injury in U.S. police-reported passenger-vehicle crashes, 2016–2024
Source: PLoS One. 2026 Nov 12;21(11):e0362075. doi: 10.1371/journal.pone.0362075 (PMC13759116; doi:10.1371/journal.pone.0362075)
Supplement: Supplementary file 19 [file pone.0362075.s019.docx]

**S16 Table. Vehicle-age associations and age heterogeneity for fatal injury.**

| **Result type** | **Group or contrast** | **aOR or ratio (95% CI)** | **F** | **df1** | **df2** | **P** | **Holm P** |
| --- | --- | --- | --- | --- | --- | --- | --- |
| Overall association | 5–9 vs 0–4 years | 1.19 (1.06–1.34) | — | — | — | — | — |
| Overall association | 10–14 vs 0–4 years | 1.62 (1.46–1.79) | — | — | — | — | — |
| Overall association | 15–19 vs 0–4 years | 2.18 (1.99–2.39) | — | — | — | — | — |
| Overall association | 20+ vs 0–4 years | 2.49 (2.27–2.73) | — | — | — | — | — |
| Age-specific association | 0–17 years: 20+ vs 0–4 | 2.21 (1.14–4.28) | — | — | — | — | — |
| Age-specific association | 18–44 years: 20+ vs 0–4 | 2.29 (1.96–2.69) | — | — | — | — | — |
| Age-specific association | 45–64 years: 20+ vs 0–4 | 2.86 (2.30–3.55) | — | — | — | — | — |
| Age-specific association | 65+ years: 20+ vs 0–4 | 2.63 (1.93–3.58) | — | — | — | — | — |
| Joint test | Complete vehicle age category × age group interaction | — | 2.83 | 12 | 42 | 0.006 | — |
| Joint test | Equality of 20+ vs 0–4 log(aOR) across age groups | — | 0.91 | 3 | 42 | 0.446 | — |
| Pairwise contrast | 0–17 vs 18–44 | 0.96 (0.46–2.00) | — | — | — | 0.915 | 1.000 |
| Pairwise contrast | 0–17 vs 45–64 | 0.77 (0.37–1.63) | — | — | — | 0.486 | 1.000 |
| Pairwise contrast | 0–17 vs 65+ | 0.84 (0.44–1.60) | — | — | — | 0.585 | 1.000 |
| Pairwise contrast | 18–44 vs 45–64 | 0.80 (0.60–1.07) | — | — | — | 0.135 | 0.809 |
| Pairwise contrast | 18–44 vs 65+ | 0.87 (0.60–1.27) | — | — | — | 0.465 | 1.000 |
| Pairwise contrast | 45–64 vs 65+ | 1.09 (0.73–1.62) | — | — | — | 0.670 | 1.000 |

Note. All 20 occupant-age-group-by-vehicle-age-category cells contained fatal events. Pairwise contrasts are ratios of the first-listed to the second-listed age-specific aORs for the ≥20-versus-0–4-year comparison.
